# Metal-Free CO Prodrugs Activated by Molecular Oxygen Protect against Doxorubicin-Induced Cardiomyopathy in Mice

**DOI:** 10.1021/acs.jmedchem.4c01431

**Published:** 2024-10-17

**Authors:** Xiaoxiao Yang, Wen Lu, Rodrigo W. Alves de Souza, Qiyue Mao, Dipak Baram, Ravi Tripathi, Gangli Wang, Leo E. Otterbein, Binghe Wang

**Affiliations:** † Chemistry Department, Center for Diagnostics and Therapeutics, Georgia State University, Atlanta, Georgia 30303, United States; ‡ Department of Surgery, Beth Israel Deaconess Medical Center, Harvard Medical School, Boston, Massachusetts 02215, United States

## Abstract

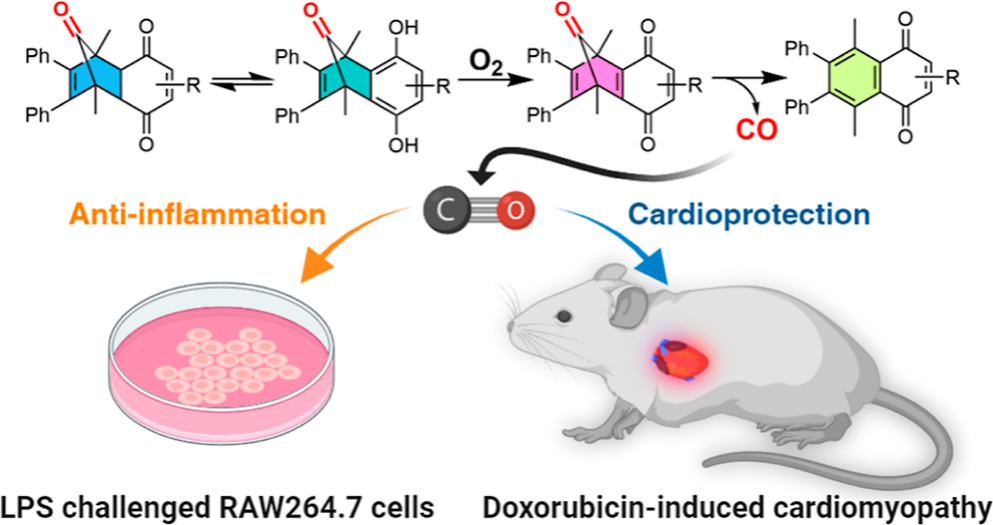

Carbon monoxide has been extensively studied for its various therapeutic activities in cell cultures and animal models. Great efforts have been made to develop noninhalational approaches for easy and controlled CO delivery. Herein, we introduce a novel metal-free CO prodrug approach that releases CO under near-physiological conditions. CO from the quinone-derived CO prodrugs is initiated by general acid/base-catalyzed tautomerization followed by oxidation by molecular oxygen to form the key norbornadienone intermediate, leading to cheletropic CO release only in an aerobic environment. Representative CO prodrug analog **QCO-105** showed marked anti-inflammatory effects and HO-1 induction activity in RAW264.7 macrophages. In a mouse model of doxorubicin-induced cardiomyopathy, we show for the first time that the CO prodrug **QCO-105** prevented cardiomyocyte injury, consistent with the known organ-protective effects of HO-1 and CO. Overall, such a new CO prodrug design serves as the starting point for developing CO-based therapy in attenuating the cardiotoxicity of doxorubicin.

## Introduction

**Figure 1:**
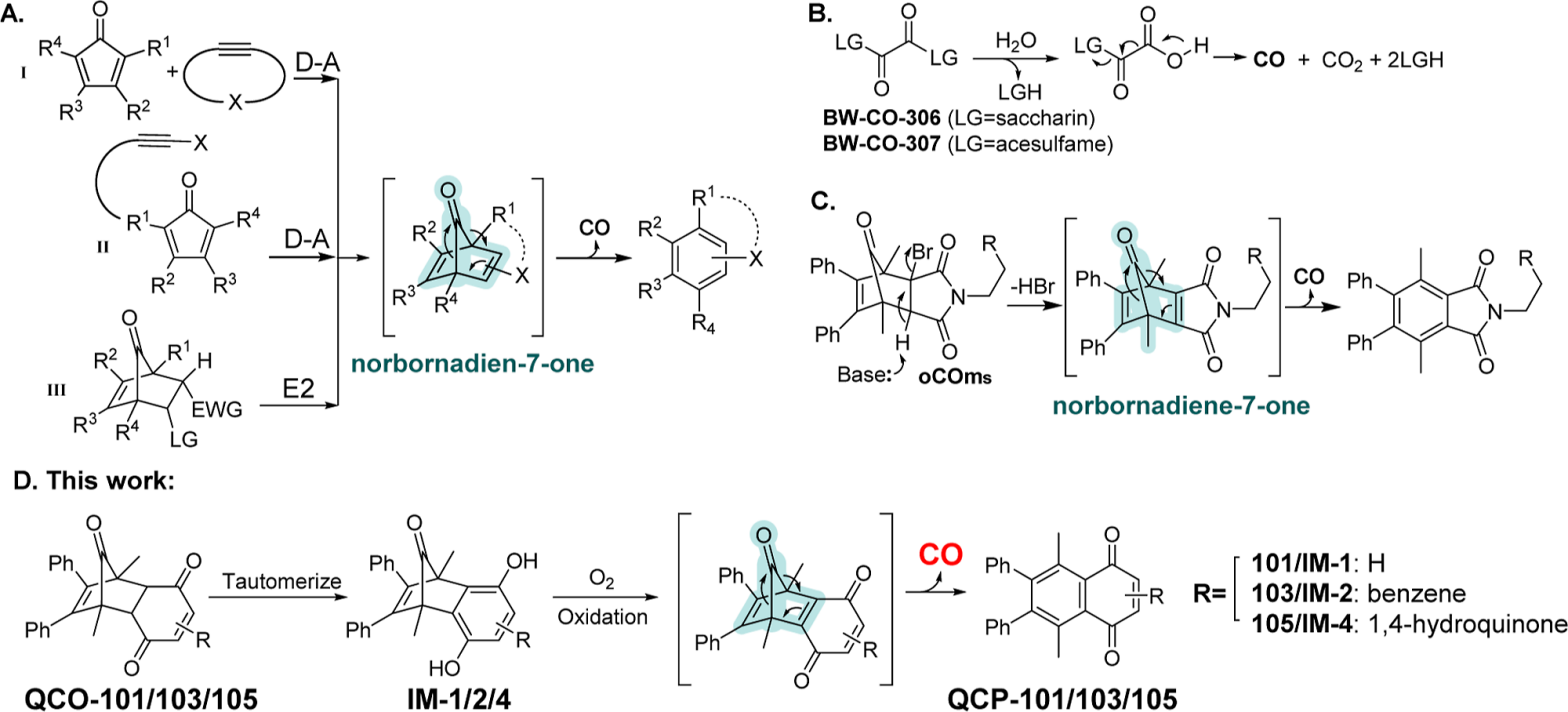
Organic CO donors capable of CO release under near-physiological conditions without light irradiation. (A) (I) Intermolecular D–A reaction-based CO prodrug; (II) intramolecular D–A reaction-based CO prodrug; (III) elimination-based CO prodrug (LG: leaving group, EWG electro-withdrawing group); (B) oxalic acid-based CO prodrugs using artificial sweeteners as “carriers”; (C) elimination-based CO donors reported by the Larson group; and (D) tautomerization-oxidation-cheletropic reaction-based CO prodrugs described in this work.

Carbon monoxide (CO) has primarily been known as a toxic gas, but this perception has changed in recent years with the wide recognition of CO’s endogenous production, pathophysiological roles, and pharmacological activities.^1^ Exogenously delivered CO regulates a number of cellular processes, including inflammation,^2^ the immune response,^3^ apoptosis,^4^ neurotransmission,^5^ as well as neuromodulation and cognition,^6^ to name a few.^1^ The possible signaling roles of endogenous CO and the therapeutic potential of exogenous CO have motivated research efforts toward developing novel chemical approaches to deliver CO for biological application. The last two decades has witnessed the development of metal-based CO-releasing molecules (CORMs)^7^ including those capable of nucleophile-, photo-, enzyme- or redox-triggered release,^7−11^ photosensitive organic CO donors,^12−14^ CO in solution,^15^ CO in ultrasound-sensitive materials,^16,17^ CO in foams,^18^ and organic CO donors capable of releasing CO under near physiological conditions.^12,19−22^ We have a long-standing interest in developing metal-free organic CO prodrugs to deliver CO^23^ and have developed a series of prodrugs by taking advantage of decarbonylation chemistry of oxalyl derivatives^24,25^ and cheletropic CO release from an unstable norbornadienone intermediate.^26,27^ In the latter case, we used bimolecular^28,29^ and unimolecular^30^ Diels–Alder reaction and elimination reactions^31−34^ to build the key norbornadienone intermediate (Figure 1). In a similar manner, Larsen’s group independently developed a series of organic CO donors using cheletropic CO release from norbornadienone formed by an elimination reaction. Such donors showed promising bioactivity in modulating vasorelaxation and organ protection.^35^ Herein, we describe a new chemistry design for activating an organic prodrug for CO release through a tautomerization-initiated reaction cascade that relies on oxidation by molecular oxygen (Figure 1D).

## Results and Discussion

### Design and Synthesis of Quinone-Based CO Prodrugs

In our continuous efforts to design new CO prodrugs, we are especially interested in approaches that use new mechanisms for prodrug activation and afford control of the selective delivery. The cheletropic reaction that forms the key norbornadiene-7-one intermediate has proven to be a preferential approach which allows for the incorporation of various trigger mechanisms including concentration (enrichment),^29^ aqueous environment,^30^ pH changes,^31^ reactive oxygen species,^32,34,36^ and enzymes.^33,37^ In the approach using a cheletropic extrusion reaction to release CO, designing an appropriate strategy to generate the norbornadiene-7-one intermediate is the key step (Figure 1A and C). Because dihydroquinones are known to undergo air-oxidation^38−40^ and oxygen is readily available in the mammalian biological environment including cell culture, blood, and upper GI tract,^41^ we have designed a prodrug approach that requires oxygen oxidation as a key step for activation. This new design with a unique mechanism of activation offers a chance of a CO release in an oxygenated environment. Similar oxygen responsiveness has been achieved with the molybdenum-based CORM (ALF186).^42^ As we opt for developing metal-free organic CO prodrugs, we sought to utilize quinone chemistry to generate the key norbornadienone intermediate for CO release (Figure 1D). Since such a process involves ketone-enol tautomerization and oxidation, we envisioned that the quinone-based CO prodrugs (QCOs) would release CO under physiological conditions with pH and oxygen dependency, which could potentially be useful to achieve some selectivity in CO delivery. As a proof-of-concept, we chose to first synthesize **QCO-101**, a known compound that had been examined in the context of the Diels–Alder reaction^43^ and Heck coupling reactions.^44^ The synthesis was accomplished through Diels–Alder reaction between cyclopentadienone **1** and benzoquinone, **2a** (Table 1). After confirming the structure of **QCO-101** by NMR, we examined whether the tautomerization of **QCO-101** would lead to the formation of norbornadien-7-one and subsequent CO release (Figure 1).

**Table 1:**
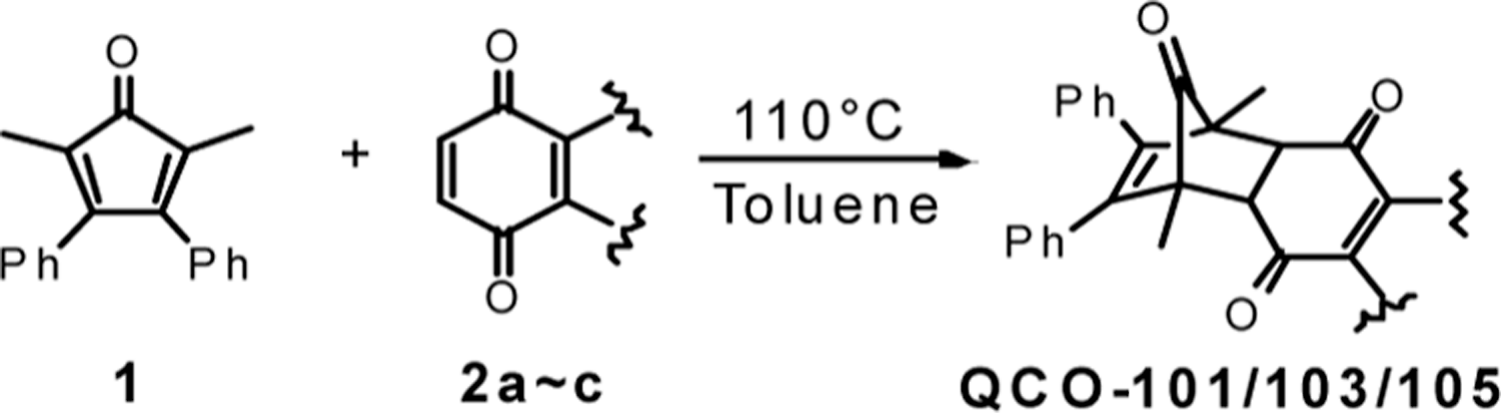

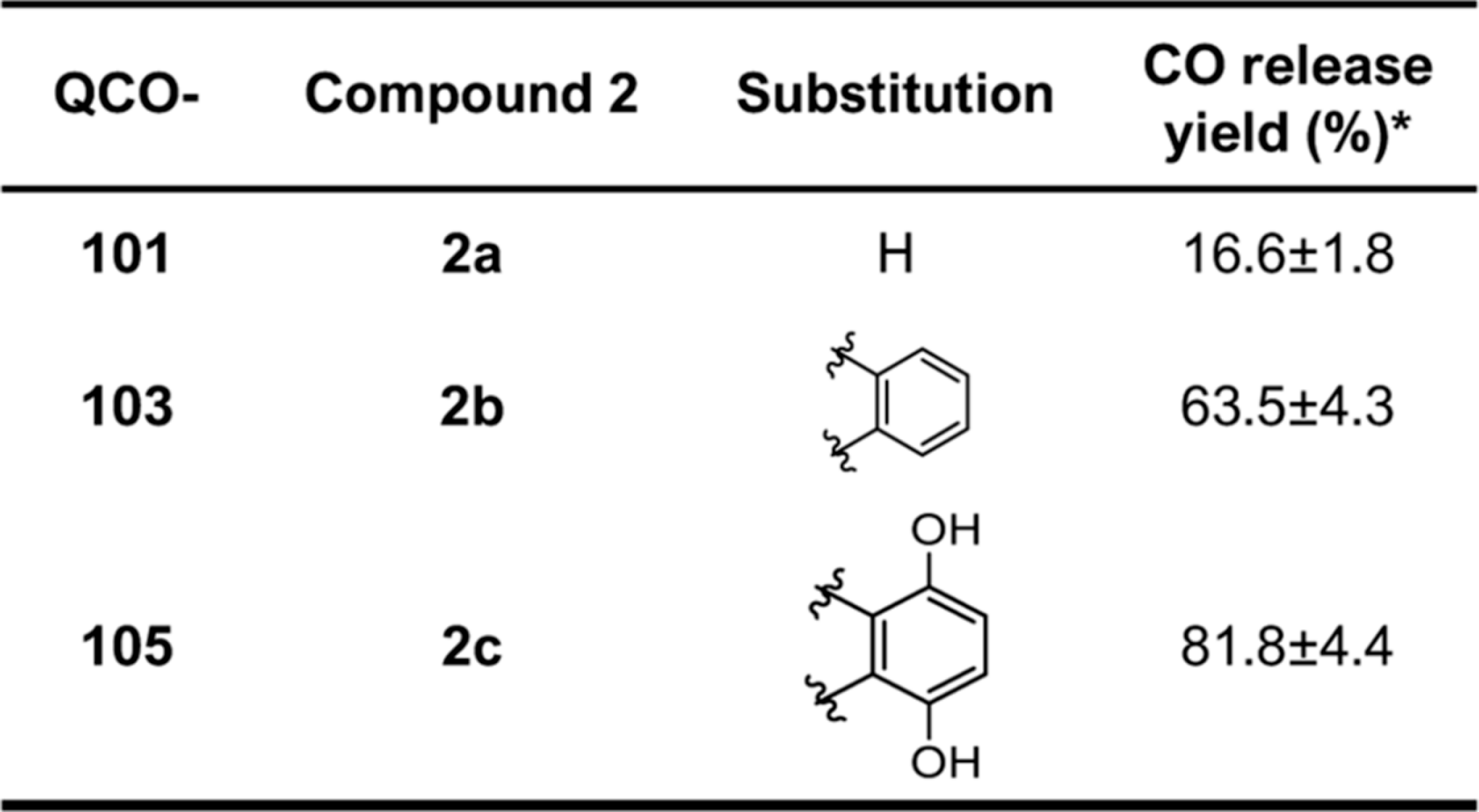
Structures and CO Release Yield

a Tested with gas chromatography (GC) in pH 7.4 PB (10 mM):DMSO = 1:9 (v/v) solution.

When **QCO-101** was dissolved in a mixed solvent of phosphate buffer (PB)/DMSO (1:9) at pH 7.4 in a headspace vial at 25 °C, CO generation was detected by GC equipped with a methanizer-coupled FID detector. However, the CO yield was only about 17% after incubation for 20 h (Table 1). The side product, naphthoquinone **QCP-101** (see the Supporting Information for details), was obtained with a yield of 18%, indicating a stoichiometric CO release. Notably, the enol intermediate (**IM-1**) was stable enough to be separated from the reaction mixture in a 67% yield (Figure 1). Interestingly, although **IM-1** technically has sp^2^-hybridized carbon atoms that might formally fulfill the norbornadiene-7-one structural feature, it did not spontaneously release CO as those generated from the Diels–Alder reaction between cyclopentadiene and alkyne (Figure 1a).^30^ This could be due to the electro-donating nature of the hydroquinone moiety, which disfavors the cheletropic extrusion reaction.^45^ Alternatively, the aromaticity of the dihydroquinone moiety might be too much of a stabilizing factor to allow for a spontaneous cheletropic reaction. There might be some interesting theoretical issues to examine. It is interesting to note that the reaction of a cyclopentadienone with a benzyne species did lead to CO generation at over 200 °C, although the cheletropic reaction involved using a “double bond” from a benzene ring as part of the “norbornadienone” moiety.^46^ It is unknown whether such a reaction would occur at room temperature. This issue may deserve additional theoretical studies.

In this study, we focused on the initial feasibility and practical issues. Though **QCO-101** succeeded in achieving partial CO release, we desired a higher CO yield under near-physiological conditions for biological application. One way to do so is to facilitate the oxidation step of the reaction. We reasoned that extending the conjugation might facilitate the oxidation step thus resulting in a decrease in aromatic stabilization, as shown in previous reports.^38,47^ As such, we expected the extended conjugation to be favorable for enhanced susceptibility toward air oxidation and thus CO release. Therefore, 1,4-naphthoquinone **2b** and naphthazarin **2c** were chosen to react with cyclopentadienone **1** to give **QCO-103** and **QCO-105**, respectively. As expected, these two new analogues showed a much higher overall CO yield in a mixed solvent of pH 7.4 PB (10 mM)/DMSO (1:9), as determined with GC (Table 1). Hence, **QCO-103** and **QCO-105** were chosen to conduct further studies.

### Factors that Impact CO-Release from QCOs

**Figure 2:**
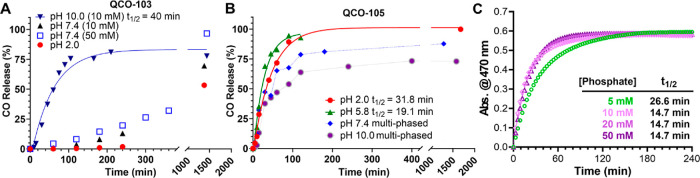
CO release profiles of QCO-103 (A) and QCO-105 (B) in PBS buffer at different concentrations. The solvent consists of 10% PB and 90% DMSO. The phosphate concentration is indicated in the figure. For (B), phosphate concentration was 10 mM for all groups. (C) UV–vis kinetics profile of **QCO-105** with designated phosphate concentrations at pH 5.8 (data interval: 3 min, [**QCO**] = 50 μM).

Because the formal reaction involves an initial tautomerization step, which is known to be pH-dependent, we next assessed the CO release profiles of **QCO-103** and **QCO-105** at different pH values using GC. As expected, the CO release kinetics and the overall yields were found to be pH-dependent (Figure 2A). Specifically, the release of CO from **QCO-103** was much faster under basic conditions (pH 10) with a half-life (*t*_1/2_) of 40 min. Under neutral (pH 7.4) or acidic (pH 2.0) conditions, the CO yield was much lower at 13% and 2%, respectively, at the 4 h point. The maximal overall CO release from **QCO-103** at 24 h was 69% at pH 7.4 and 53% at pH 2.0. Such results support a pH effect on the enolization step or the oxidation step of the reaction cascade.^48^ We further probed the effect of the buffer concentration at pH 7.4 to see whether the reaction involved general acid/base catalysis. Specifically, we studied the CO release rate in 10 and 50 mM PB and found a noticeably faster reaction with a higher CO yield at 50 mM PB. Such results indicate that general acid/base catalysis in the cascade reaction of **QCO-103** is most likely at the enolization step.

**QCO-105** was examined in a similar fashion and was found to have a more desirable CO release profile at pH 7.4 (lysosome), pH 5.8 (lysosome), and pH 2.0 (gastric fluid). Specifically, in a mixed solvent of pH 5.8 PBS (10 mM):DMSO (1:9), the *t*_1/2_ of CO release was 19.1 min with almost quantitative CO release in 2 h (Figure 2B). At pH 2.0, quantitative CO release was achieved with a *t*_1/2_ value of 38.1 min. Proton NMR studies using deuterated TFA/D_2_O as the acidic condition also showed complete conversion of **QCO-105** to **QCP-105** in 16 min without noticeable formation of an intermediate (Supporting Information, Figure S5). The kinetic profiles at higher pH (pH 7.4 or pH 10) showed a fast initial phase (0–30 min) followed by a leveling-off phase (30–120 min), with the overall CO release yield being 82% and 70% at pH 7.4 and 10 in 10 mM PBS, respectively. Such results suggest the existence of a non-CO-releasing pathway under more basic conditions. The color of the CO-release reaction mixture of **QCO-105** changed from light yellow to orange. Therefore, we monitored the reaction with UV–vis spectroscopy at pH 5.8 and different phosphate concentrations. When comparing the results at 5- and 10 mM PB, a slower CO release rate (Figure 2C) was observed at 5 mM compared to the reaction at 10 mM. The dependency of CO release on the buffer concentration indicates general acid/base catalysis. However, further increasing the buffer concentration to 20–50 mM did not lead to additional increases in the reaction rate, giving an initial indication of a switch in the rate-determining step (RDS) of the reaction cascade (Figure 2C). By analyzing UV spectra (Supporting Information Figure. S1) and the CO release profile, the following three conclusions can be made. First, UV spectra (Figure S1) do not show perfect isosbestic point(s). Such results are in agreement with the proposed mechanism for the reaction sequence occurring through a stable intermediate, which accumulates to a small extent. Second, the isosbestic point(s) is more coherent at a low 5 mM PB concentration (Supporting Information, Figure. S1), indicating that enolization is the rate-limiting step under such conditions with no accumulation of an additional intermediate. However, the imperfection of the isosbestic point(s) became more pronounced at higher concentrations of the PB, indicating the accumulation of an intermediate under such conditions and possibly a switch of the RDS to either the oxidation step or a subsequent step. Considering the fast cheletropic extrusion reaction rate (*k*_3_ on the 10^4^ ∼ 10^5^ s^–1^ scale according to literature^49^) for the last step, it is reasonable to suggest the RDS not being the cheletropic step. Data in Figure 2C appear to show the formation of the final product to follow pseudo-first-order reaction kinetics with a half-life of 26.6 min under these conditions (5 mM, 37 °C) at pH 5.8 with a rate constant *k*_1_ of ∼4.16 × 10^–4^ s^–1^. This value is generally on the same scale as literature tautomerization reaction kinetics tested with broadband dielectric spectroscopy.^50,51^ To the best of our knowledge, our results represent the first case of using a simple UV–vis method to examine tautomerization kinetics by studying these quinone derivatives under near-physiological conditions. Third, when the phosphate concentration was increased to 10 mM, the overall reaction kinetics increased, consistent with a general acid/base-catalyzed reaction in which proton transfer is involved in the RDS. Increasing the phosphate concentration further did not change the apparent pseudo-first-order reaction half-lives, which were around 14.7 min (Figure 2C, Supporting Information, Figure S1). Such results suggest a switch of the rate-limiting step to the oxidation reaction at buffer concentrations higher than 10 mM. Such a deduction is supported by the appearance of the more pronounced intermediate peak (presumably the enol species) in the UV–vis spectra around 500 nm. LC–MS analysis confirmed the appearance of the enol intermediate in the sample (Figure S2). Therefore, the oxidation reaction pseudo-first-order rate constant under the specific experimental conditions we used ([PB] = 10–50 mM) can also be calculated as *k*_2_’ ≈ 8.3 × 10^–4^ s^–1^. To this end, we show that CO release from **QCO-103** and **QCO-105** can be readily achieved under near-physiological conditions and that the CO release kinetics is pH-dependent and follows a general acid/base catalysis mechanism.

### CO Release Mechanism Studies

**Scheme 1:**
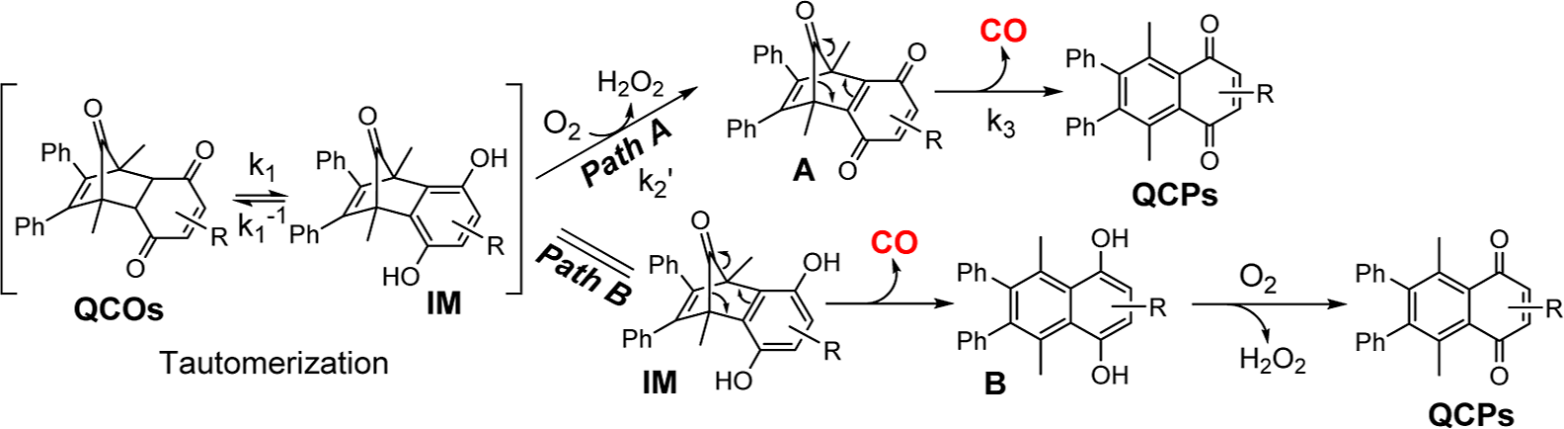
Proposed Mechanism of Tautomerization-Induced CO Generation

To further examine the proposed reaction mechanism (Figure 1 and Path A in Scheme 1), we compared it against the other plausible pathway (Path B, Scheme 1). In pathway A, cheletropic CO release happens after oxidation of hydroquinone **IM**, leading to the formation of norbornadienone intermediate **A**. However in pathway B, CO release occurs via direct cheletropic extrusion of CO from the tautomer **IM** to form the intermediate **B**, which undergoes oxidation to form **QCP** products.

**Figure 3:**
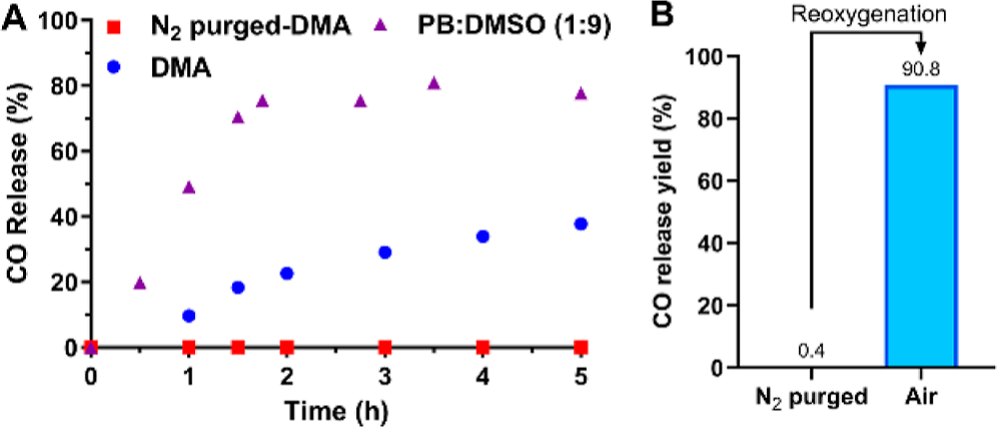
CO release profile in a controlled oxygen environment measured by GC. (A) **QCO-103** was incubated in N_2_ purged DMA, normal DMA, and PB/DMSO (1:9, v/v, pH 10, 10 mM); (B) **QCO-105** was incubated in N_2_ purged PB/DMSO (1:9, v/v, pH 5.8, 10 mM) for 2 h followed by GC analysis of headspace gas. Afterward, 1 mL of air was injected into the headspace vial followed by incubation for 2 h and GC analysis.

The successful separation of relatively stable intermediate **IM** from the CO release reaction of **QCO-101** serves as a strong initial indication of Pathway A being the most likely mechanism. It is still essential to study whether oxidation is a prerequisite for CO release, especially for **QCO-103** and **QCO-105** which are structurally analogous, but different from **QCO-101**. First, we analyzed the CO release reaction of **QCO-103** using HPLC. Dimethylacetamide (DMA) was used to replace the PBS/DMSO mixture as a solvent to slow down the reaction. In pure DMA, the CO release rate was much slower than that in the PB (pH 10):DMSO mixture (Figure 3A), presumably owing to the slower enolization in DMA compared with a buffered aqueous solution. Nevertheless, when deoxygenated DMA was used in a N_2_ prepurged reaction in a two-compartment setting (see Supporting Information Section 2.3 for details), no CO release was detected by GC analysis (Figure 3A). Such results demonstrate the essential nature of oxidation by molecular oxygen for the release of CO release. We interpret these results as indicating pathway A is the dominant CO release pathway for **QCO-103** to release CO. Such a deduction is also supported by further HPLC and LC–MS studies (Supporting Information, Figures S3 and S4) of the same reaction conditions, which showed accumulation of the enol intermediate (**IM-2**, Figure 1D) under oxygen-free conditions. In contrast, this intermediate remained at a minimum level under oxygenated conditions (Figure S3). Similarly, the release of CO from **QCO-105** was also found to be oxygen-dependent. Specifically, with a two-compartment setup, we confirmed that **QCO-105** did not release CO in an oxygen-free solution of a PB (pH 5.8)/DMSO (1:9) mixture. After reoxygenation by injecting 1 mL of air into the headspace vial and incubating for 30 min, GC analysis showed a 91% CO release after 2 h (Figure 3B). It should be noted that air oxidation of **IM** generates hydrogen peroxide as a side product. However, because of the presence of the ubiquitous catalase in mammalian cells and its effective decomposition of hydrogen peroxide at a rate (*k*_cat_ = 3.8 × 10^7^ s^–1^, *k*_cat_/*k*_m_ = 3.5 × 10^7^ M^–1^ s^–1^)^52^ much faster than its generation from **IM** (*ca*. 8.3 × 10^–4^ s^–1^, this study), we anticipate minimal effect of the peroxide generated from **IM**.^53^ Experimentally, we have indirect evidence to support this point. Though externally added H_2_O_2_ is known to induce HO-1 expression,^54^**QCO-103** did not induce HO-1 despite generating H_2_O_2_ to a similar extent as **QCO-105**_._ As a result, we did not further pursue the issue of H_2_O_2_ generation from **IM**. To further study the CO release chemistry, we also confirmed the two-electron transfer oxidation reaction process with an electrochemistry study (Supporting Information, Figure S10), which is consistent with the proposed CO-release mechanism. To this end, we show that pathway A is the most likely reaction mechanism for the release of CO from these three CO prodrugs.

### CO Release in Cell Culture Models

**Figure 4:**
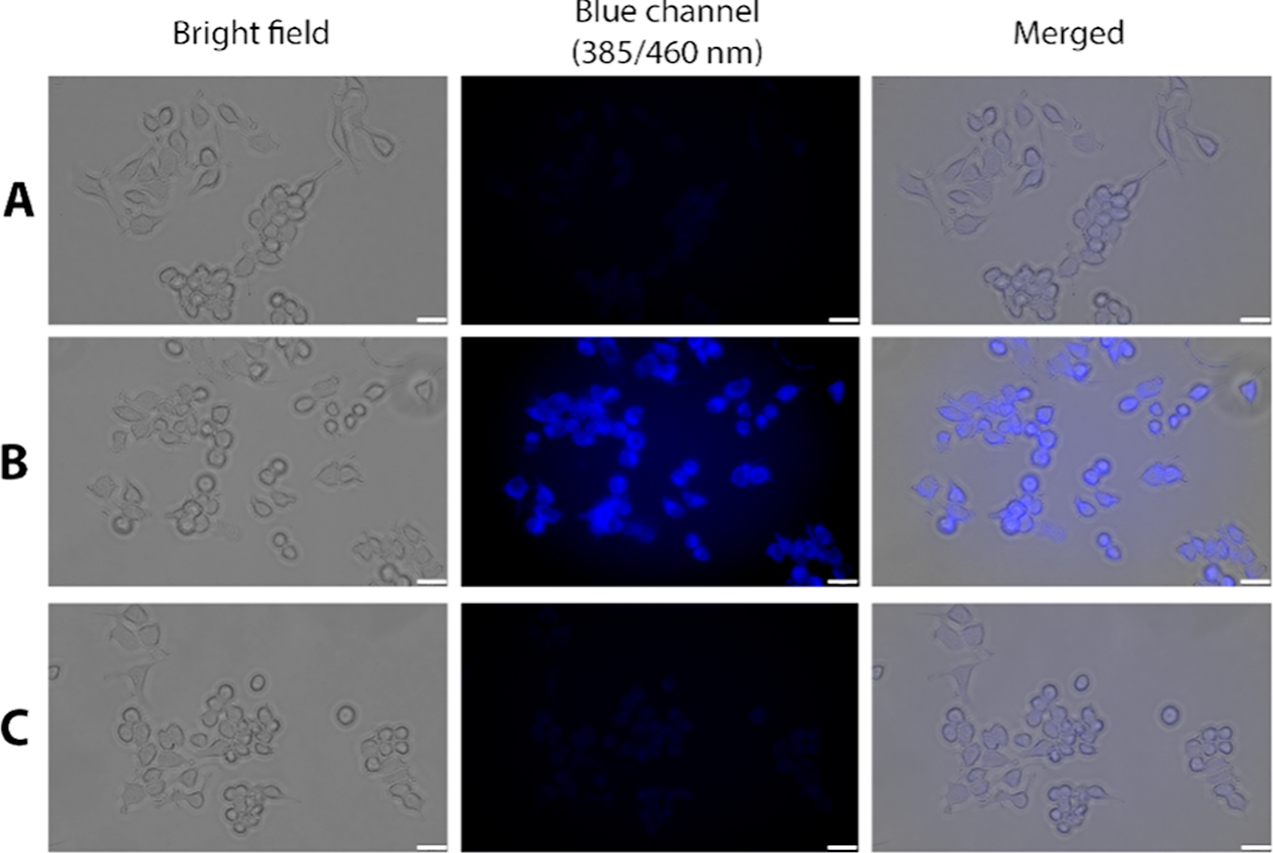
Cell imaging of CO using the CO probe CODP-202 in macrophages in response to QCO-103 and QCP-103. RAW264.7 cells incubated with (A) 10 μM CODP-202 for 60 min; (B) 10 μM **CODP-202** plus 50 μM **QCO-103** for 60 min; and (C) 10 μM **CODP-202** plus 50 μM **QCP-103** for 60 min; scale bar: 25 μm.

After studying the CO release chemistry, we next studied whether **QCO-103** and **QCO-105** release CO in cell culture and exhibit anti-inflammatory activity, as observed with other CO prodrugs we have developed.^23,26,30,34,55^ First, we used a CO probe (**CODP-202**) developed in our laboratory^56^ to detect whether **QCO-103** generated CO in cell culture. After the incubation of RAW264.7 cells with 50 μM **QCO-103** and 10 μM **CODP-202** for 60 min, significant blue fluorescence was seen, confirming CO production (Figure 4). In contrast, neither vehicle control nor **QCP-103** showed noticeable fluorescence. We conducted the same experiments using a second CO probe, COP-1,^57^ with the same conclusion of CO release (Supporting Information, Figure S6).

**Figure 5:**
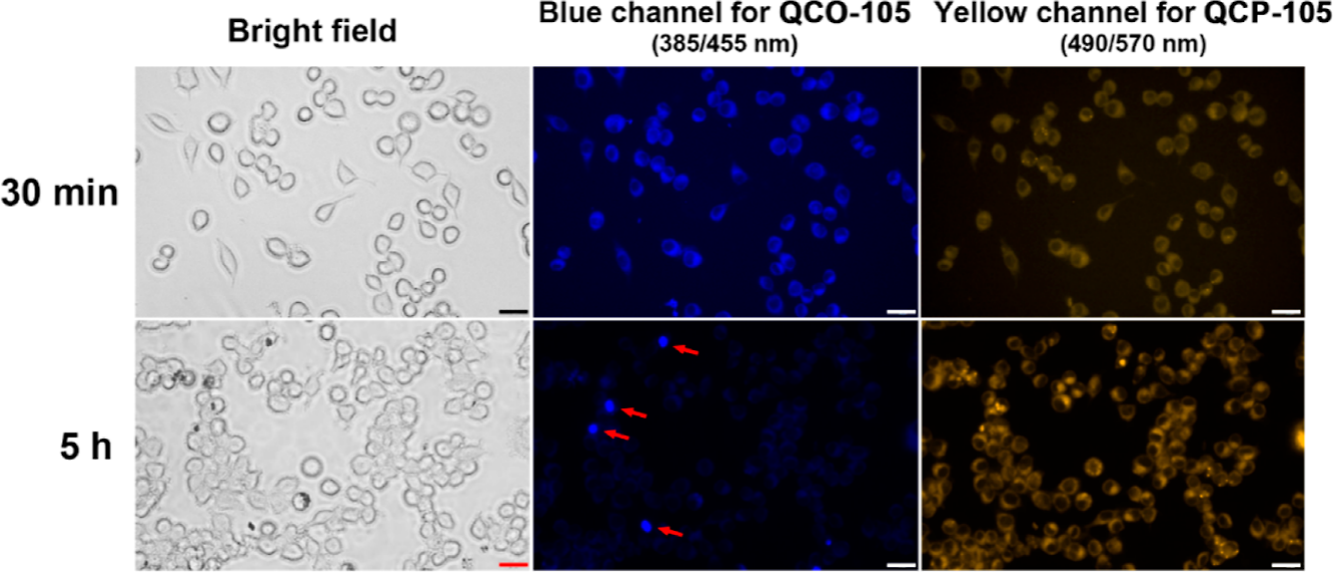
Transformation of **QCO-105** to **QCP-105** in RAW264.7 cells (red arrows point to precipitated **QCO-105**; scale bar: 25 μm).

To further study the conversion of **QCO-105** in cell culture, we took advantage of the inherent fluorescence of **QCO-105** and **QCP-105** (Supporting Information, Figure S7). Specifically, **QCO-105** elicits blue fluorescence with a maximum excitation and emission wavelength of 385 and 435 nm, respectively. Upon conversion to **QCP-105** after CO release, the maximum emission wavelength is red-shifted to 552 nm, yielding a yellow fluorescence. These characteristic fluorescence properties allowed for visualizing the transformation of **QCO-105** to **QCP-105** in a cell culture using fluorescence microscopy. Specifically, after incubating RAW264.7 cells with **QCO-105** for 30 min, blue fluorescence was detected in the cells while the yellow channel showed weak fluorescence (Figure 5). After further incubation for 5 h, blue fluorescence significantly decreased, while strong yellow fluorescence emerged, indicating conversion of **QCO-105** to **QCP-105** intracellularly. It must be noted that due to the low aqueous solubility of **QCO-105**, several fluorescent particles remained in images of the blue fluorescence channel, likely due to the incomplete conversion of undissolved **QCO-105** to **QCP-105**. Such results support the deduction that solubilized **QCO-105** released CO in the cell culture. Collectively, these data show the conversion of **QCO-103** and **QCO-105** to their respective **QCP** products with simultaneous release of CO in RAW264.7 macrophages.

### Anti-inflammatory Activity of QCO-103 and QCO-105 in Macrophages

**Figure 6:**
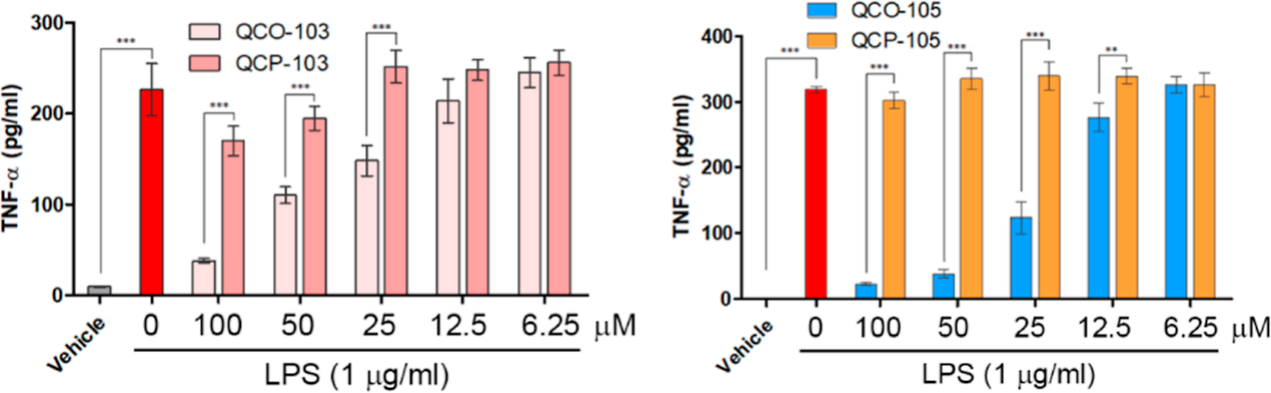
Anti-inflammatory effects of **QCO-103** and **QCO-105**. RAW264.7 cells were pretreated with QCO and QCP compounds at different concentrations for 5 h, followed by the addition of LPS (1 μg/mL) and incubation for 1 h. Culture medium containing 0.5% DMSO was used as the vehicle control (*n* = 3, **P < 0.01, ***P < 0.001, *t*-test, TNF is measured by ELISA after 5× dilution of the culture medium).

CO is known to exert anti-inflammatory activity both in vitro and in vivo.^7^ Lipopolysaccharide (LPS)-induced release of TNF from RAW264.7 cells has been extensively used as an in vitro model to evaluate the anti-inflammatory activity of CO prodrugs and CO gas.^24,30^ Therefore, we next tested the anti-inflammatory activity of compounds **QCO-103/-105** and their CO-released product **QCP-103/-105** by ELISA. As shown in Figure 6, LPS induced a significant increase in TNF expression in RAW264.7 cells while preincubation with **QCO-103** or **QCO-105** for 5 h, followed by LPS, significantly inhibited TNF expression in a dose-dependent manner. Incubation with control compounds **QCP-103/-105** had no effect on LPS-induced TNF. Such results are consistent with CO as the anti-inflammatory component of **QCO-103** and **QCO-105**. Cytotoxicity assays showed no effect of the compounds on cell viability within the period of the assay (Figure S8).

### QCO-105 Induces HO-1 Expression in Macrophages

**Figure 7:**
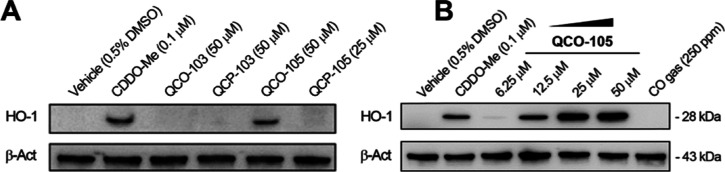
**QCO-105** dose-dependently increases the level of HO-1 expression in RAW264.7 macrophages, which was not observed in response to **QCO-103** or **QCP-105**. (A) QCO and QCP at 25 and 50 μM for 6 h; (B) **QCO-105** dose-dependently induced HO-1 expression at 6 h (CDDO-Me was used as the positive control).

It has been reported that a conjugate of an Mn-based CORM and Nrf2 activator dimethylfumarate, HYCO-3, showed CO release in cells as well as stimulation of HO-1 expression.^58^ It is also known that some naturally occurring anthraquinone derivatives such as rhein^59^ and rubrofusarin,^60^ also increase HO-1 expression, thus offering cytoprotective and/or anti-inflammatory activities.^61^ We next tested whether **QCO-103** and **QCO-105** would induce HO-1 (Figure 7A). Interestingly, **QCO-105** at 25 μM significantly induced HO-1 expression in a dose-dependent fashion comparable to CDDO-Me as a positive control (Figure 7B).^62^ Neither **QCO-103** nor **QCP-105** induced HO-1 expression despite being administered at a higher concentration. It is worth noting that 250 ppm of CO gas did not induce HO-1 expression in RAW264.7 cells, as we recently reported,^63^ indicating that the induction of HO-1 by **QCO-105** is likely due to a CO-independent mechanism.

It is well-known that exposure to CO or increasing HO-1 expression suppresses TNF expression in macrophages challenged with LPS.^64,65^ The HO-1/CO axis also protects against TNF-mediated inflammation in the pancreas, liver,^66^ and lungs^67^ through the Nrf2-ARE–HO–1 axis. HO-1 activation offers protective effects in the liver,^68^ intestine,^69^ kidney,^70^ and heart,^71^ to name a few.^72^ Therefore, CO generation coupled with HO-1 activation may render **QCO-105** a good candidate for treating systemic inflammation and organ injury.

### QCO-105 Treatment Prevents Doxorubicin-Induced Cardiotoxicity in Mice

**Figure 8:**
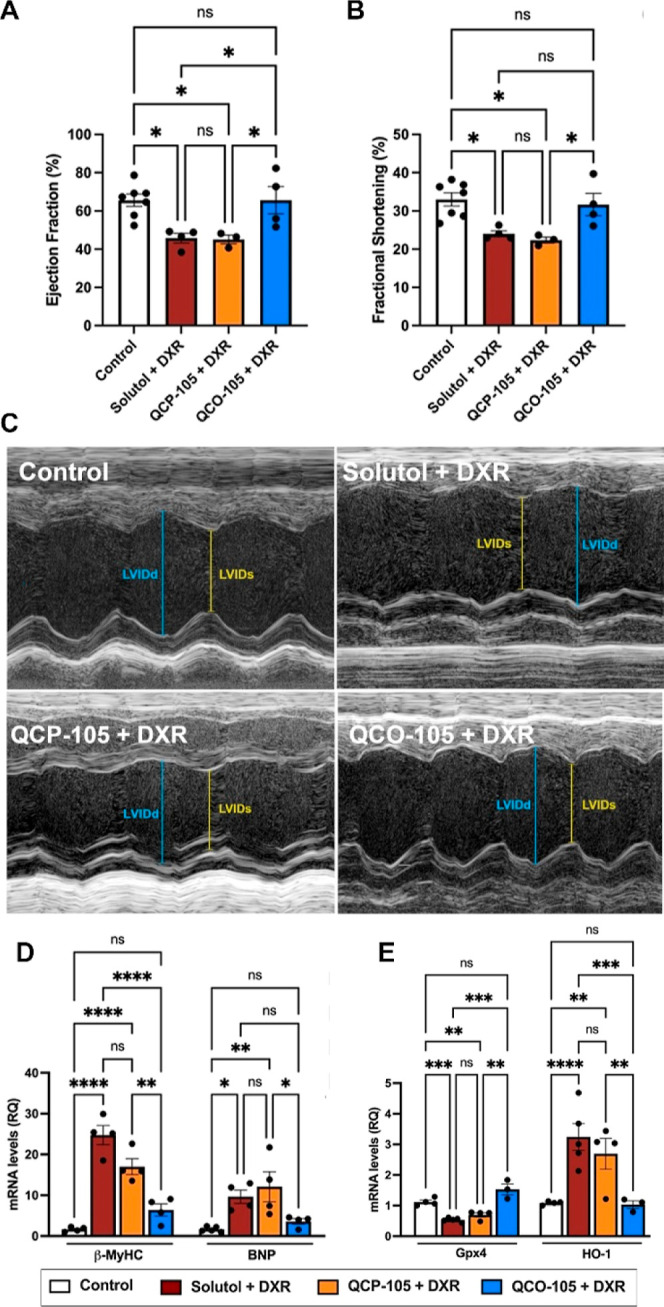
QCO-105 prevents acute doxorubicin (DXR) toxicity to the heart and attenuates the expression of markers of DXR-induced ferroptosis. QCO-105 (50 mg/kg, ip) attenuated DXR (20 mg/kg)-induced cardiac dysfunction after 7 days, evidenced by preserved ejection fraction (A) and fractional shortening (B); (C) representative echocardiography (LVIDd: left ventricular internal dimension in diastole, LVIDs: left ventricular internal dimension in systole); (D) QCO-105 prevented DXR-induced upregulation of the cardiac dysfunction markers β-MyHC and BNP compared to QCP and Solutol controls (3–7 mice per group); and (E) Gpx4 and HO-1 mRNA expression in the ventricle of mice 7 days after a single DXR injection *±* QCO-105 (3–5 mice per group, Solutol was used as the vehicle). Expression was determined by RT-qPCR and normalized to that of Hprt1. Mean ± SEM; ns: no significant difference, *n* ≥ 3, *P < 0.05, **P < 0.01, ***P < 0.001, and ****P < 0.0001.

Among all the protective effects of exogenously administered CO or endogenous CO generated through increased HO-1, we were interested in studying the cardioprotective effects of the CO prodrugs.^71,73^ Our work and others have demonstrated the potent cytoprotective effects of exogenous CO in the heart.^74−77^ This occurs in part, by preventing cardiomyocyte death and promoting overall cardiovascular health.^71,73,78^ Previous findings have demonstrated the benefits of inhaled and oral CO against DXR-induced cardiotoxicity.^79,80^ We also observed that pretreatment with **QCO-105** protected against DXR-induced toxicity in H9c2 mouse cardiomyocytes (Supporting Information, Figure S9). Given the promising CO/HO-1 dual function of **QCO-105**, we were interested in testing whether the prophylactic administration of **QCO-105** would protect the heart against acute DXR-induced cardiac dysfunction in mice. Specifically, **QCO-105**, **QCP-105**, and Solutol vehicle alone were administered to separate cohorts of mice 1 h before injecting a single dose of DXR (20 mg/kg, i.p.). Cardiac function was examined both before and 7 days after DXR administration by echocardiography. DXR induces highly reproducible cardiomyopathy in mice, which is a very well-characterized clinically relevant model. Administration of **QCO-105** significantly attenuated DXR-induced cardiac dysfunction, evidenced by a preserved ejection fraction and fractional shortening compared to **QCP** and vehicle (Solutol) controls (Figure 8A–C). Subsequently, we investigated the impact of **QCO-105** on stress response genes in the heart in response to DXR, specifically examining β-MyHC (beta-myosin heavy chain), a gene known to be upregulated with cardiac failure or hypertrophy, and B-type natriuretic peptide (BNP), a marker of cardiac dysfunction.^81^ We observed that **QCO-105** effectively prevented DXR-induced upregulation of β-MyHC and BNP mRNA compared to that of the vehicle (Solutol) and **QCP-105**-treated mice (Figure 8D). Collectively, the ability of **QCO-105** to preserve cardiac function upon prophylactic administration indicates its potential as a cardioprotective agent against DXR-toxicity.

### QCO-105 Mitigates Markers of DXR-Induced Ferroptosis

Although the detailed cytoprotective mechanism of **QCO-105** and CO gas is yet to be fully understood, existing research indicates that CO can prevent cell death.^73,82^ Notably, ferroptosis is a recently identified form of programmed cell death that has been implicated in the development of DXR-induced cardiomyopathy.^83^ Ferroptosis is primarily driven by the excessive accumulation of pro-oxidant iron in cells, leading to lipid peroxidation and protein damage. Because the HO-1/CO axis is closely involved in iron metabolism, we next measured the expression of glutathione peroxidase 4 (Gpx4) and HO-1 in the heart on day 7 after DXR administration. Gpx4 is a key antioxidant enzyme that protects against lipid peroxidation and is a hallmark of ferroptosis.^83,84^ Mice treated with DXR show a >50% decrease in Gpx4 in the heart, which was completely prevented by treatment with **QCO-105** (Figure 8E). Neither Solutol nor **QCP-105** had any effect on DXR-induced reduction in Gpx4 expression in the heart, suggesting that CO prevents cardiac dysfunction in part by preserving antioxidative capacity against lipid peroxidation and therefore ferroptosis.

DXR is also known to upregulate HO-1 expression in the heart, contributing to ferroptosis.^83^ There are paradoxical interpretations of such DXR-induced HO-1 expression. On the one hand, HO-1 is a stress-response element and its upregulation by DXR is part of the acute stress response to limit cell and tissue injury.^83^ Recent work shows that the knockdown of HO-1 in the heart impacts mitochondrial quality control; aggravates the cardiac response to ischemia/reperfusion injury and promotes fibrosis.^71,85−87^ On the other hand, activation and upregulation of HO-1 is indicative of DXR-induced ferroptosis and cardiomyopathy.^83^ When HO-1 expression was measured in the heart on day 7 after DXR treatment, it was found that DXR led to elevated HO-1 expression (Figure 8E), indicative of ongoing injury more than any beneficial effects of HO-1 induction. In contrast, the **QCO-105** treatment showed normal HO-1 expression in the heart. While the DXR-**QCP-105** treatment showed HO-1 expression in the heart equivalent to DXR-Solutol-treated controls. Such combined findings reflect the ability of CO to limit cellular injury and the overall stress response in vivo in this acute injury model.^83,84^ Collectively, our observations suggest that **QCO-105** may hold the potential to mitigate DXR-induced myocardial injury in part through mechanisms that involve preventing the loss in antioxidant power, as indicated by the preservation of Gpx4 levels and blocking the upregulation of BNP and β-MyHC. Much more mechanistic work in cell culture and animal models is needed to elucidate the detailed molecular signaling events, but the ability of **QCO-105** to offer cardioprotection in mouse models justifies additional work with this class of CO prodrugs in organ protection.

## Conclusions

In our continuous efforts to explore the therapeutic potential of developing “CO in a pill”, we are searching for novel CO prodrugs with unique molecular mechanisms for selective cellular and tissue targeting. In this study, we describe a unique structural class of CO prodrugs activated by molecular oxygen through a cascade reaction including enolization, oxidation, and cheletropic CO release. Furthermore, such prodrug activation is both pH- and O_2_-dependent. The mechanism by which CO is released from these compounds (**QCO-103/-105**) has been carefully examined through various studies. These two prodrugs suppress inflammation as evidenced by their ability to inhibit LPS-induced TNF production in macrophages. This directed us to test the pharmacological efficacy of **QCO-105** in a mouse model of doxorubicin-induced cardiotoxicity as DXR toxicity is in part driven by a robust inflammatory reaction that occurs in the heart after administration. A very pronounced cardioprotective effect was observed in mice treated with **QCO-105** prior to administration of cardiotoxin DXR. DXR is an effective chemotherapy with applications from leukemia and Hodgkin’s lymphoma to cancers affecting the breast, stomach, lung, thyroid, bladder, and soft tissue. However, it can have significant harmful effects on the heart both acutely and chronically,^88^ which remains an unaddressed medical need. The mechanism (s) of action of **QCO-105** is likely through its ability to generate CO, which in turn regulates the expression of anti-inflammatory/antioxidant genes such as HO-1, and inhibition of ferroptosis. Other signaling pathways such as the recently reported heme-dependent Ca^2+^ sensitization effect of CO in cardiomyocytes using an organic CO prodrug may also contribute to the overall cardioprotective activity of CO.^89^ Taken together, the cardioprotective effects recapitulate what has been observed with inhaled CO and CORM.^79^ As a result, the development of this family of CO prodrugs warrants further mechanistic and pharmacological explorations for the treatment of DXR toxicity and other diseases of the heart.

## Experimental Section

### General

Chemical reagents were purchased from Sigma-Aldrich (Saint Louis, Missouri, USA) and Oakwood (Estill, South Carolina, USA). Solvents were purchased from Fisher Scientific (Pittsburgh, Pennsylvania, USA), and the dry solvents were prepared by a Vigor Tech purification system (Houston, Texas, USA). Silica gel for the flash column was purchased from Sigma-Aldrich (St. Louis, Missouri, USA). Certificated CO calibration gas was purchased from GASCO (Oldsmar, Florida, USA). Flash column separation was conducted on a Biotage SP1 system (Charlotte, North Carolina, USA). HPLC analysis was performed on an Agilent 1100 HPLC system (Santa Clara, California, USA). ^1^HNMR (400 MHz) and ^13^CNMR (101 MHz) were analyzed with Bruker AV-400 MHz Ultra Shield NMR. Data for ^1^HNMR and ^13^C NMR are reported in terms of chemical shift (δ, ppm). Multiplicity [s = singlet, d = doublet, t = triplet, q = quartet, m = multiplet or unresolved, br = broad singlet, coupling constant(s) in Hz]. The fluorescence spectrum was recorded on a Shimadzu RF-5301PC fluorospectrometer (Kyoto, Japan). GC was analyzed using Agilent 7820A system with Restek 5A mole sieve column (2m, 0.53 mm ID), Restek CH4izer methanizer coupled with FID. All compounds were >95% pure by HPLC analysis or elemental analysis.

### General Procedure to Synthesize QCO Compounds

In a 20 mL scintillation vial equipped with a magnetic stirring bar was weighed 100 mg (192 mmol) of 2,5-dimethyl-3,4-diphenylcyclopentadienone dimer and 384 mmol quinone derivatives: **QCO-101**: benzoquinone; **QCO-103**: 1,4-naphthoquinone; and **QCO-105**: naphthazarin. The reagent was dissolved in 10 mL of toluene, and the reaction was stirred at 110 °C for 5 h. The solvent was evaporated in vacuo, and the residue was purified by flash column using ethyl acetate/hexane as the eluent. The target fraction was collected and evaporated to dryness to yield the target compound.

#### 1,4-Dimethyl-2,3-diphenyl-1,4,4a,8a-tetrahydro-1,4-methanonaphthalene-5,8,9-trione **(QCO-101)**

Yield %: 82%, white powder. ^1^H NMR (400 MHz, CDCl_3_, δ): 7.20–7.11 (m, 6H), 6.85 (s, 2H), 6.81 (dd, *J* = 6.5, 2.9 Hz, 4H), 3.30 (s, 2H), 1.54 (s, 6H). ^13^C NMR (101 MHz, CDCl_3_, δ): 199.9, 195.8, 143.9, 141.7, 133.2, 129.5, 128.4, 127.9, 59.0, 52.0, 11.9. HRMS–ESI: 391.1310 calcd for C_25_H_20_O_3_Na; [M + Na]^+^ found, 391.1324.

#### Synthesis of Tautomerized Intermediate 5,8-Dihydroxy-1,4-dimethyl-2,3-diphenyl-1,4-dihydro-1,4-methanonaphthalen-9-one **(IM-1)**

**IM-1** was separated from the CO-release reaction mixture of **QCO-101** in a DMSO solution after lyophilization by flash chromatography. ^1^H NMR (400 MHz, CDCl_3_, δ): 7.21–7.18 (m, 6H, benzene Ar–H), 7.07–7.05 (m, 4H, benzene Ar–H), 6.52 (s, 2H, hydroquinone Ar–H), 4.78 (br-s, 2H, phenol-OH), 1.82 (s, 6H, –CH_3_). Due to stability concerns, residual solvents (EtOAc/Hexane) were not completely evaporated. ^13^C NMR (101 MHz, CDCl_3_, δ): 194.4, 148.1, 145.6, 134.5, 131.7, 130.0, 129.3, 128.4, 128.2, 127.8, 115.9, 60.4, 10.7. HRMS–ESI: 391.1310 calcd for C_25_H_20_O_3_Na; [M + Na]^+^ found, 391.1325.

#### 1,4-Dimethyl-2,3-diphenyl-1,4,4a,9a-tetrahydro-1,4-methanoanthracene-9,10,11-trione **(QCO-103)**

Yield %: 76%, white powder. ^1^H NMR (400 MHz, CDCl_3_, δ): 8.00 (dd, *J* = 5.8, 3.3 Hz, 2H), 8.00 (dd, *J* = 5.8, 3.3 Hz, 2H), 7.70 (dd, *J* = 5.8, 3.3 Hz, 2H), 7.06 (dd, *J* = 8.3, 6.3 Hz, 2H), 7.00 (t, *J* = 7.4 Hz, 4H), 6.45 (d, *J* = 7.2 Hz, 4H), 3.53 (s, 2H), 1.61 (s, 6H). ^13^C NMR (101 MHz, CDCl_3_, δ): 200.1, 194.5, 141.5, 137.2, 134.5, 133.2, 129.5, 128.0, 127.6, 127.5, 59.5, 53.1, 12.1. HRMS–ESI: 419.1467 calcd for C_29_H_23_O_3_ [M + H]^+^ found, 419.1638.

#### 5,8-Dihydroxy-1,4-dimethyl-2,3-diphenyl-1,4,4a,9a-tetrahydro-1,4-methanoanthracene-9,10,11-trione **(QCO-105)**

Yield %: 72%, yellow powder. ^1^H NMR (400 MHz, CD_3_CN, δ): 12.23 (s, 2H), 7.30 (s, 2H), 7.18–7.04 (m, 6H), 6.63–6.52 (m, 4H), 3.63 (s, 2H), 1.54 (s, 6H). CD_3_CN was chosen due to the overlapping of the signal for aromatic protons with CDCl_3_. ^13^C NMR (101 MHz, CDCl_3_, δ): 200.2, 200.0, 156.5, 141.9, 133.2, 129.2, 129.2, 128.3, 127.8, 115.9, 59.7, 52.4, 12.3. HRMS–ESI: 473.1365 calcd for C_29_H_22_O_5_Na; [M + Na]^+^ found, 473.1358.

#### General Procedure to Synthesize CO-Released QCP Compounds

QCO compounds were dissolved in DMSO and heated at 60 °C overnight with an oxygen balloon. After the reaction completion was checked by TLC, the reaction was lyophilized to remove DMSO. The residue was further purified by flash chromatography if necessary.

#### 5,8-Dimethyl-6,7-diphenylnaphthalene-1,4-dione **(QCP-101)**

Yield %: 18%, yellow solid. ^1^H NMR (400 MHz, CDCl_3_, δ): 7.16–7.09 (m, 6H), 6.94–6.78 (m, 6H), 2.41 (s, 6H). ^13^C NMR (101 MHz, CDCl_3_, δ): 188.2, 148.9, 140.0, 138.8, 137.9, 131.2, 129.6, 127.8, 126.8, 20.4. HRMS–ESI: 361.1204 calcd for C_24_H_18_O_2_Na; [M + Na]^+^ found, 361.1205.

#### 1,4-Dimethyl-2,3-diphenylanthracene-9,10-dione **(QCP-103)**

Yield %: 85%, yellow powder. ^1^H NMR (400 MHz, CDCl_3_, δ): 8.17 (dd, *J* = 5.7, 3.3 Hz, 2H), 7.74 (dd, *J* = 5.7, 3.3 Hz, 2H), 7.13 (dq, *J* = 14.5, 7.2 Hz, 6H), 6.91 (d, *J* = 7.0 Hz, 4H), 2.50 (s, 6H). ^13^C NMR (101 MHz, CDCl_3_, δ): 186.8, 148.9, 140.2, 138.1, 134.8, 133.5, 133.2, 129.7, 127.8, 126.7, 126.5, 20.9. HRMS–ESI: 411.1361 calcd for C_28_H_20_O_2_Na; [M + Na]^+^ found, 411.1374.

#### 5,8-Dihydroxy-1,4-dimethyl-2,3-diphenylanthracene-9,10-dione **(QCP-105)**

Yield %: 92%, orange powder. ^1^H NMR (400 MHz, CDCl_3_, δ): 12.97 (s, 2H), 7.27 (s, 2H), 7.18–7.09 (m, 6H), 6.92–6.90 (m, 4H), 2.54 (s, 6H). ^13^C NMR (101 MHz, CDCl_3_, δ): 190.2, 156.6, 149.8, 140.0, 139.5, 132.8, 129.6, 128.3, 127.9, 126.9, 114.0, 21.8. HRMS–ESI: 443.1259 calcd for C_28_H_20_O_4_Na; [M + Na]^+^ found, 443.1268.

### HPLC and LCMS Analysis

Column: Kromasil C18 5 μm, 4.6 × 150 mm. Mobile phase: A: 0.1% TFA in H_2_O; B, 0.1% TFA in ACN. Flow rate: 1 mL/min. Gradient: 5–95% B (0–10 min), 95% B (10–12 min), 95% to 5% B (12–12.1 min), 5% B (12.1–15 min); Detector: DAD monitored at 220 and 254 nm. LCMS analysis was performed on AB Sciex API 3200 LC–MS/MS (ESI) system (Framingham, Massachusetts, USA) with Agilent 1200 HPLC serving as the LC module. Column: Waters SunFire C18 3.5 μm, 3 × 150 mm. Mobile phase: A: 0.1% FA in H_2_O; B: 0.1% FA in ACN. Flow rate: 0.5 mL/min. Gradient: 5–95% B (0–10 min), 95% B (10–12 min), 95% to 5% B (12–12.1 min), 5% B (12.1–15 min).

### Cell Culture

RAW 264.7 and H9c2 cells were purchased from the American Type Culture Collection (ATCC, Manassas, VA, USA). Raw 264.7 and H9c2 cells were cultured in Dulbecco’s modified Eagle’s medium (DMEM, Corning) supplemented with 10% fetus bovine serum (FBS, Corning) and 1% penicillin/streptomycin (PNS). Fetal bovine serum (FBS), DMEM, and Trypsin–EDTA (0.05%) were purchased from Gibco BRL (Gaithersburg, Maryland, USA). CO gas treatment was conducted under 37 °C with a gastight chamber (2500 mL volume, Mitsubishi Gas Chemical, Japan) equipped with needle valve gas inlet and outlet ports under constant supplement (5 mL/min) of premixed CO gas (250 ppm of CO, 5% CO_2_ in balanced air, Airgas, PA, USA). Cell incubator: VWR symphony (Radnor, Pennsylvania, USA). Cell counting kit-8 (CCK-8) was purchased from Dojindo (Kumamoto, Japan) and used according to the manufacturer’s manual. Optical density (OD) and microplate fluorescence assay were measured with a multiwavelength plate reader: PerkinElmer Vector3 (Shelton, Connecticut, USA). For the CCK assay, OD was measured at 450 nm; for fluorescence reading, fluorescence intensity (counts per second, CPS) was measured with the excitation filter wavelength of 405 nm and excitation filter wavelength of 535 nm, bandwidth = 10 nm, aperture: normal, signal collection time: 2 s per well. Cell imaging was conducted on an Olympus IX73 inverted fluorescence microscope (Tokyo, Japan).

### Murine Model of Cardiac Injury

10-to-12 week-old male C57BL/6 mice (Jackson Laboratories) were housed under specific pathogen-free conditions with 12 h day/light cycles. All mouse procedures were approved by the Beth Israel Deaconess Medical Center, Boston, MA, Institutional Animal Care and Use Committee (#083–2021). Briefly, **QCO-105** and **QCP-105** were tested in combination with DXR. One hour after dosing with **QCO**- or **QCP-105** (50 mg/kg, p.o., formulated with Solutol), or Solutol control, animals were infused with a single dose of DXR (20 mg/kg, i.p.; ∼60 mg/m^2^; 3–7 mice per group). Hearts of the mice treated with the acute toxicity regimen were harvested 7 days after DXR administration. The presence of DXR-induced cardiomyopathy was confirmed by mRNA quantification of the cardiac dysfunction markers (B-type Natriuretic Peptide, BNP: Nppb; and beta Myosin Heavy Chain, β-MyHC: Myh7) and measurement of cardiac function by echocardiography compared with control Solutol-treated mice (see Supporting Informatio 2.9.2). The ventricles were carefully collected, frozen in liquid nitrogen, and stored at −80 °C until analysis.

### Statistical Analysis

All data were presented as the mean ± standard deviation (*n* ≥ 3). Statistical analysis was performed by Student’s *t*-test for comparison between two groups or by one-way ANOVA for comparison among multiple groups using GraphPad Prism 9 (San Diego, CA, USA). A *P*-value of less than 0.05 was considered statistically significant. The significance level is noted in the figure legend.

## Supporting Information Available

Additional materials and methods and experimental details including UV–vis spectrum, fluorescent spectrum, GC study, LCMS study, electrochemistry study, HPLC traces, elemental analysis, Western-blot, cytotoxicity assay, animal study, and NMR spectrum of compounds (PDF)
Molecular formula strings (CSV)

## Abbreviations

ANOVA: analysis of variance
ARE: antioxidant responsive element
BNP: B-type Natriuretic Peptide
β-MyHC: beta-myosin heavy chain
CCK-8: cell counting kit-8
CDDO-Me: Bardoxolone Methyl
CO: carbon monoxide
CORM: CO-releasing molecule
DMA: dimethylacetamide
DXR: doxorubicin
Gpx4: glutathione peroxidase 4
HO-1: heme oxygenase-1
Nrf2: nuclear factor erythroid 2-related factor 2
PB: phosphate buffer
RDS: rate-determining step
ROS: reactive oxygen species
