# Supplementary material for: Metal-Free CO Prodrugs Activated by Molecular Oxygen Protect against Doxorubicin-Induced Cardiomyopathy in Mice
Source: J Med Chem. 2024 Oct 17;67(21):18981–92. doi: 10.1021/acs.jmedchem.4c01431 (PMC11571113; doi:10.1021/acs.jmedchem.4c01431)
Supplement: Supplementary file 1 — jm4c01431_si_001.pdf [file jm4c01431_si_001.pdf]

Supporting Information

Metal-free CO Prodrugs Activated by Molecular Oxygen Protect against Doxorubicin-induced Cardiomyopathy in Mice

Xiaoxiao Yang, [#,a] Wen Lu, [#,a] Rodrigo W. Alves de Souza,[b] Qiyue Mao,[a] Dipak Baram,[a] Ravi Tripathi,[a] Gangli Wang,[a] Leo E. Otterbein,*[, b] and Binghe Wang[*,a]

[a.] Department of Chemistry and Center for Diagnostics and Therapeutics, Georgia State University, Atlanta, GA 30303, USA

[b.] Department of Surgery, Beth Israel Deaconess Medical Center, Harvard Medical School, Boston, MA 02215, USA.

[#] These authors contributed equally to this work

* Corresponding authors:
Dr. Leo E. Otterbein, Email: lotterbe@bidmc.harvard.edu; Dr. Binghe Wang, Email: bwang31@gsu.edu

Table of Contents

	Page No.
**1. Supplemental figures**	**S2-S5**
Figure S1, S2 ………	S2
Figure S3………	S3
Figure S4 ………	S4
Figure S5, S6………	S5
Figure S7, S8, S9………	S6
**2. Materials and Methods**………	**S7-S10**
2.1 General information of reagents and instruments………	S7
2.2 LCMS study of **QCO-103** under controlled oxygen environment………	S7
2.3 GC study of **QCO-103** and **QCO-105** under controlled oxygen environment by two-compartment setting	S8
2.4 Electrochemistry details with Figure S10 and Table S1………	S8
2.5 ELISA assay for TNF-α level in RAW264.7 cell culture………	S9
2.6 Cell imaging with CO probes CODP-202 and COP-1………	S9
2.7 Western-blot of HO-1 in RAW264.7 cells and H9c2 cells………	S9
2.8 Cytotoxicity assay to assess the protective activity of **QCO-105** in H9c2 cells ………	S10
2.9 **QCO-105** Treatment for Doxorubicin-induced cardiotoxicity ………	S10-S11
**4. NMR spectrum**………	**S13-S17**
**5. LC-MS chromatograph of QCO-105 reaction mixture.**………	**S18-S21**
**6. Elemental analysis and HPLC trace of QCO-105 and QCP-105** ………	**S22-S23**
**7. References**………	**S24**

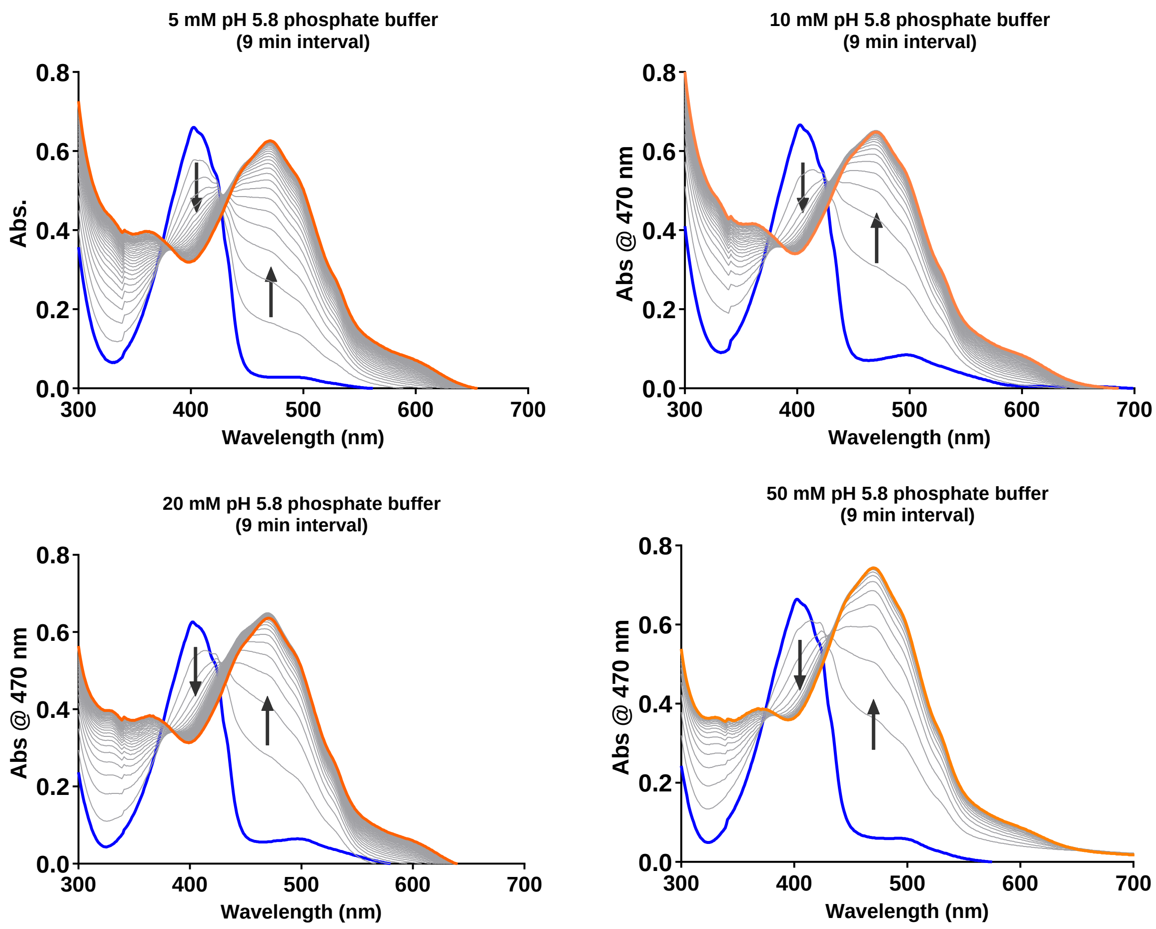

Figure S1. UV-Vis spectrum of **QCO-105** incubated in pH 5.8 phosphate buffer with various phosphate concentrations (5-50 mM).

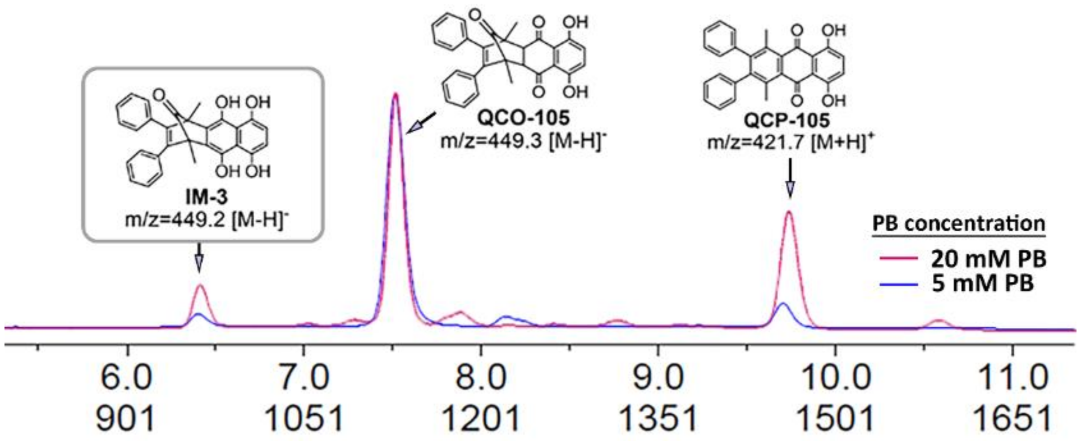

Figure S2. LC-MS results of **QCO-105** incubated in pH 5.8 phosphate buffer with different phosphate concentrations (magenta: 20 mM, blue: 5 mM). After the addition of PB buffer (100 µL) into DMSO (900 µL) solution of **QCO-105** (*c.a.* 2 mM) the reaction was incubated at 20℃ for 15 min then 5 µL of the reaction was injected into LC-MS. The

chromatograph was normalized to the largest peak of **QCO-105**. (Enol intermediate shows at t_r=6.4 min; original LC-MS data is provided in section 5 at the end of supporting information)

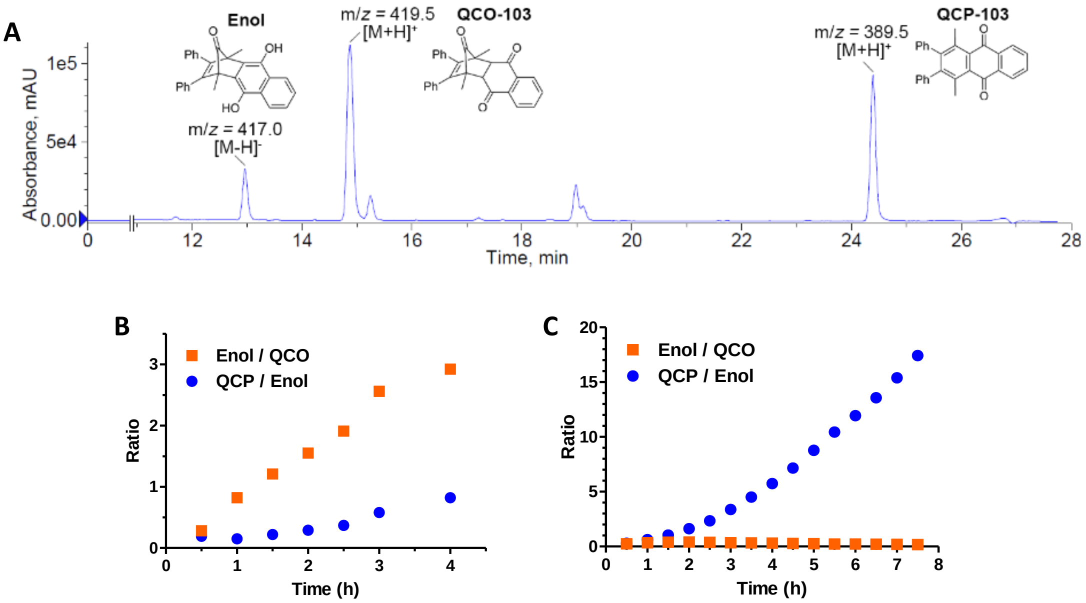

Figure S3. HPLC studies of **QCO-103** under controlled oxygen enviroment. (A) HPLC of the reaction mixture in air: peak was identified by LCMS study using the same LC conditions (see figure S3). Reaction carried out under oxygen free condtions (B) or normal air conditions (C). The areas of the peaks corresponding to **QCO-103** (QCO), the enol intermediate (Enol) and the CO-released product (QCP) were integrated. The ratio of the different species was plotted against the time. In pannel B, Enol/QCO ratio increased due to the tautomerization reation and insufficient oxidative consumption of the enol intermediate; the slow increase of QCP/Enol ratio was due to the air leakage by repeated sampling from the same reation vial. Under normoxic conditions (C), **QCP-103** was predominantly formed while the enol/QCO ratio remained lower than 0.4, indicating the oxidation reaction was fast and the enolization reaction was the rate determining step for the CO release reaction.

A. LC-MS showing the enol intermediate of **QCO-103**.

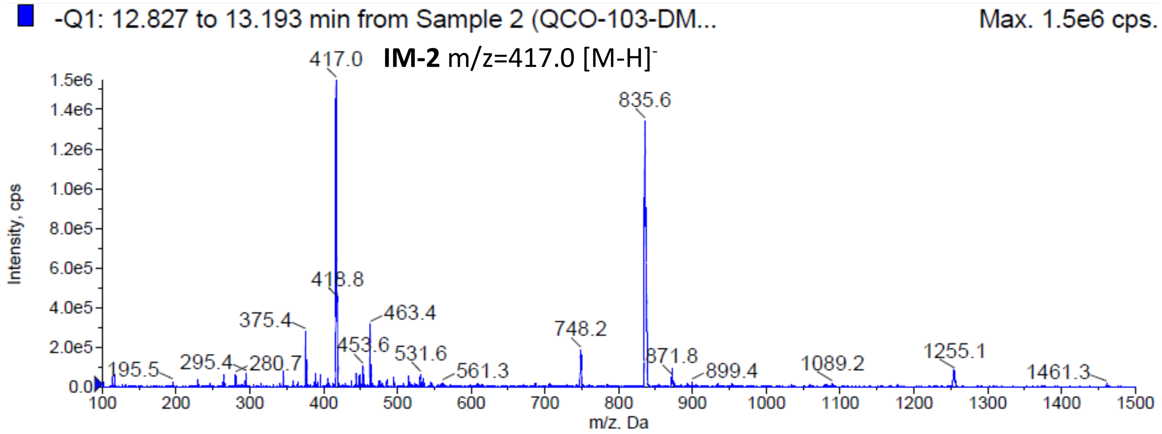

B. LC-MS showing **QCO-103**.

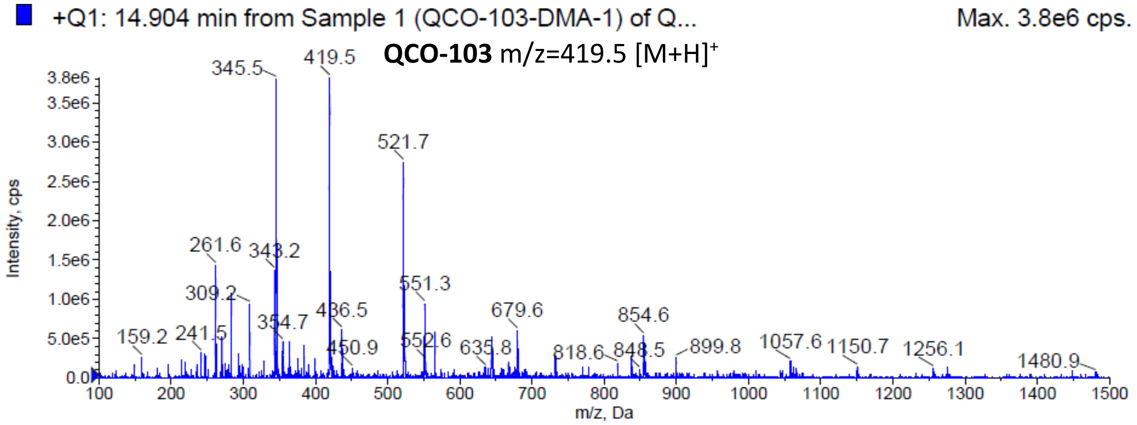

C. LC-MS showing **QCP-103**.

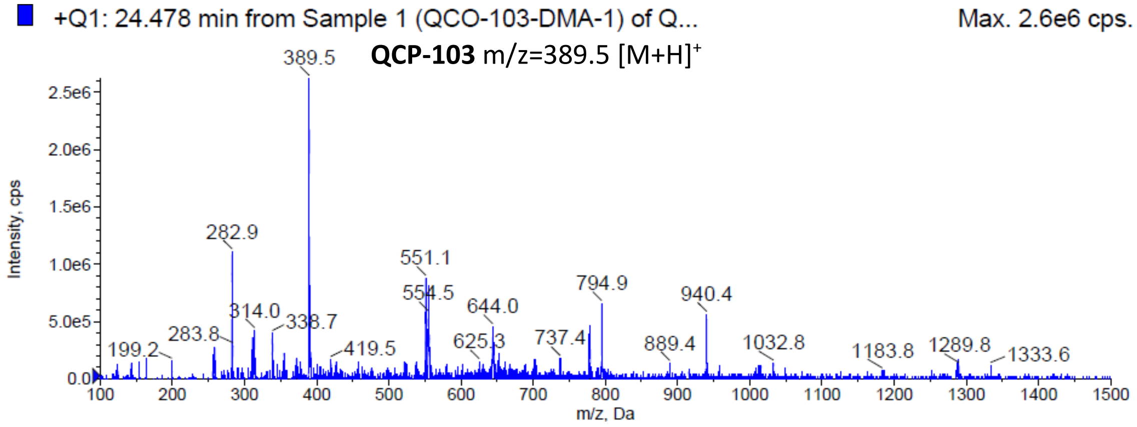

Figure S4. LCMS results of the reaction mixture of **QCO-103** in DMA. (A) Enol intermediate; (B) **QCO-103**; (C) **QCP-103**.

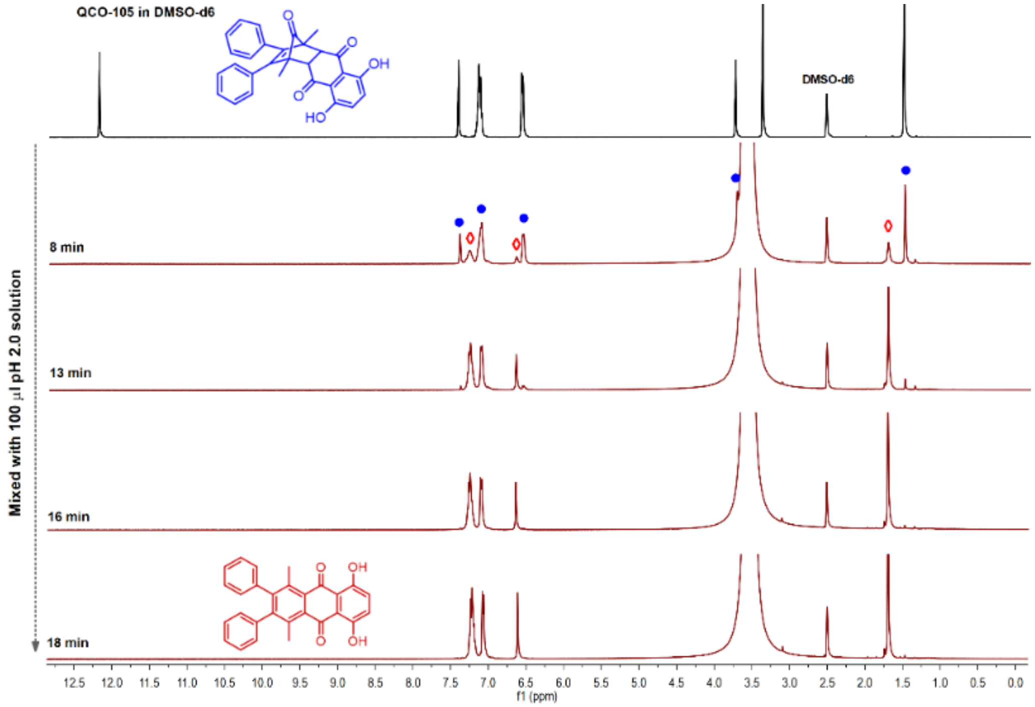

Figure S5. 1H-NMR studies of CO release reaction of **QCO-105** at pD 2 in deuterated water/TFA-d/DMSO-d6 solution showing complete conversion from **QCO-105** to **QCP-105** in 18 min.

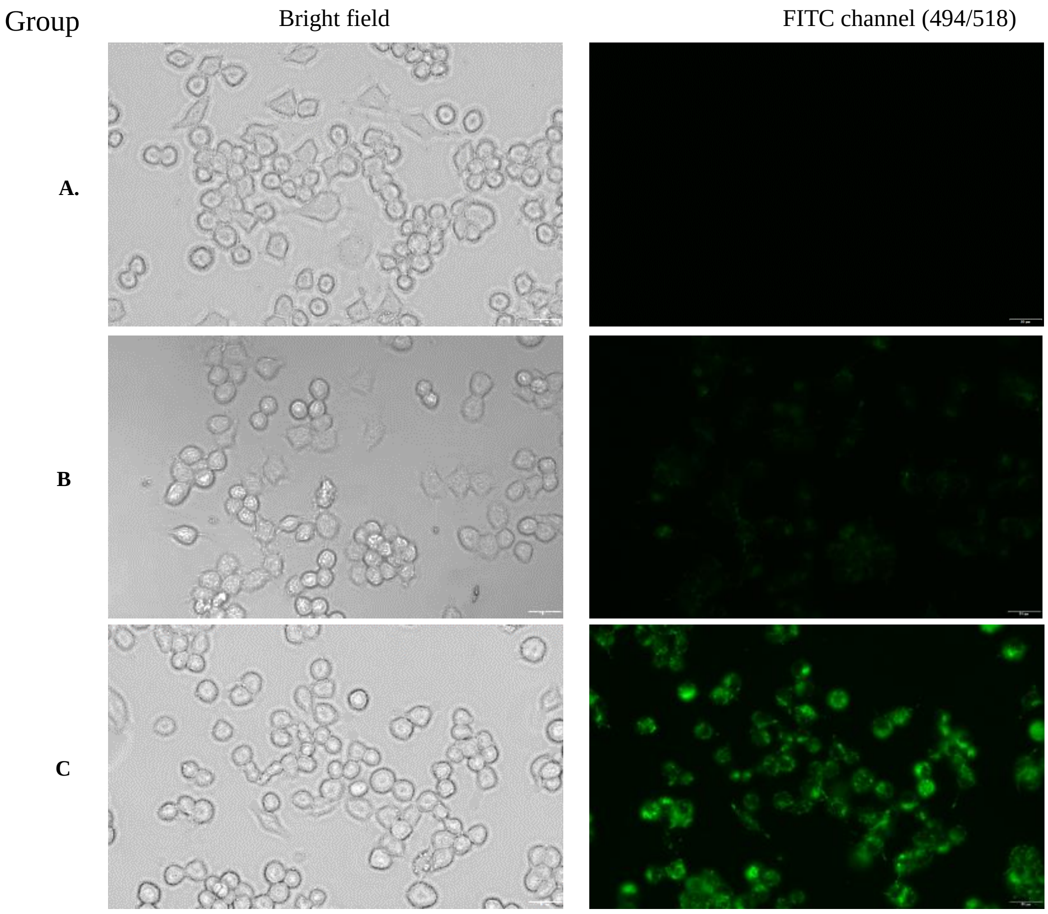

Figure S6. CO release in RAW264.7 cells detected by COP-1. (A) Raw264.7 cells incubated with 5 µM COP-1 for 2 h; (B) Raw264.7 cells incubated with 5 µM COP-1 and 50 µM **QCP-103** for 2 h; (C) RAW264.7 cells incubated with 5 µM COP-1 and 50 µM **QCO-103** for 2 h.

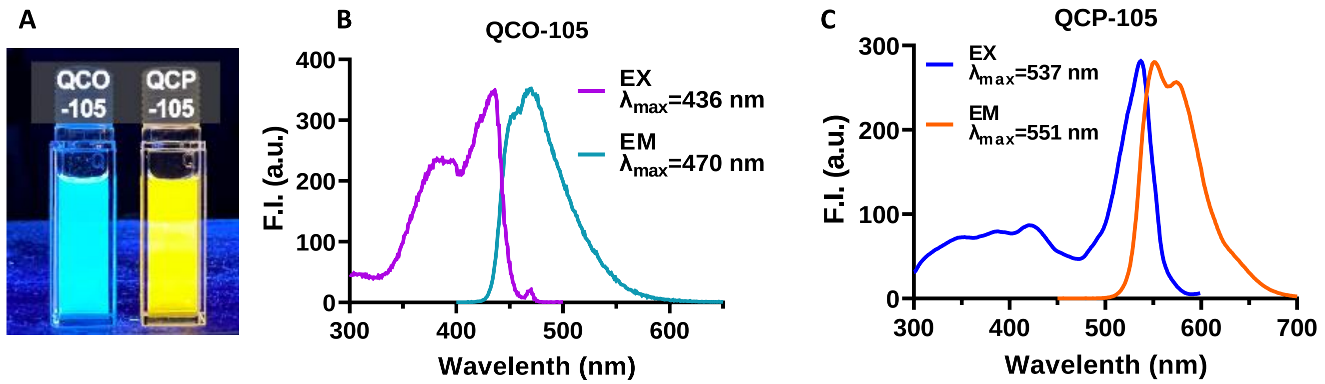

Figure S7. (A) Fluoresence image of **QCO-105** and **QCP-105** under 365 nm UV light; (B) spectra of **QCO-105** and (C) **QCP-105** in DCM.

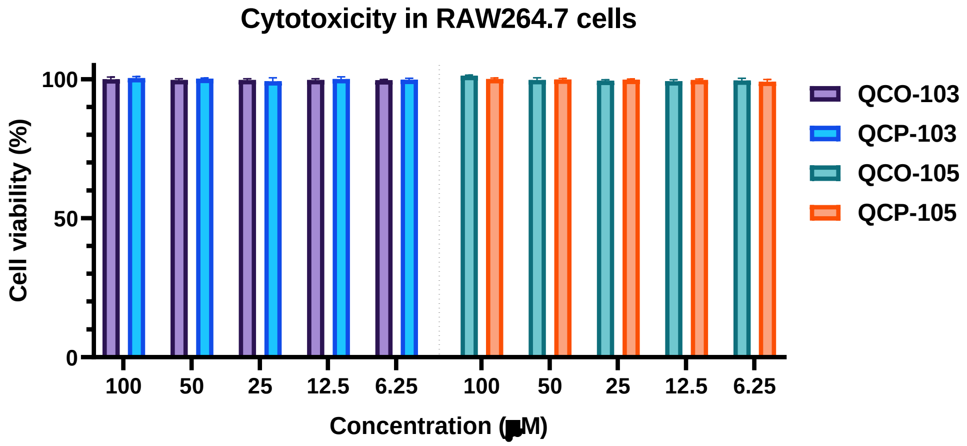

Figure S8. Cytotoxicity of **QCO-103**, **QCO-105**, **QCP-103**, and **QCP-105** in RAW264.7 cell with 6-h incubation.

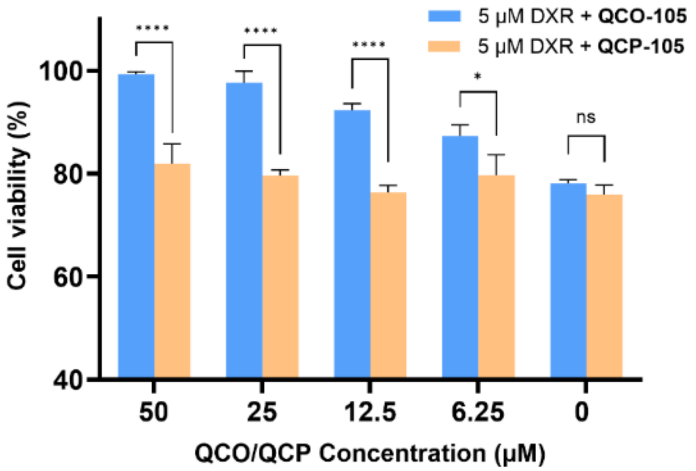

Figure S9. QCO-105 dose-dependently protects H9c2 cells from 5 μM DXR induced cytotoxicity (n=3, *P<0.05, ****P<0.0001, ns: not significant).

2. Material and Methods

2.1 General Information of Reagents and Instruments

Chemistry*:* Chemical reagents were purchased from Sigma-Aldrich (Saint Louis, Missouri, USA) and/or Oakwood (Estill, South Carolina, USA). Solvents were purchased from Fisher Scientific (Pittsburgh, Pennsylvania, USA) and the dry solvents were prepared by Vigor Tech purification system (Houston, Texas, USA). Silica gel for flash column was purchased from Sigma-Aldrich (Saint Louis, Missouri, USA). Certificated CO calibration gas was purchased from GASCO (Oldsmar, Florida, USA). Flash column separation was conducted on Biotage SP1 system (Charlotte, North Carolina, USA) HPLC analysis was performed on Agilent 1100 HPLC system (Samta Clara, California, USA). Column: Kromasil C18 5µm, 4.6 × 150 mm. Mobile phase: A: 0.1% TFA in H_2O; B: 0.1% TFA in ACN. Flow rate: 1 mL/min. Gradient: 5% to 95% B (0 - 10 min), 95% B (10 – 12 min), 95% to 5% B (12 – 12.1 min), 5% B (12.1 – 15 min); Detector: DAD monitored at 220 nm and 254 nm. LCMS analysis was performed on AB Sciex API 3200 LC-MS/MS (ESI) system (Framingham, Massachusetts, USA) with Agilent 1200 HPLC serving as the LC module. Column: Waters SunFire C18 3.5µm, 3 × 150 mm. Mobile phase: A: 0.1% FA in H_2O; B: 0.1% FA in ACN. Flow rate: 0.5 mL/min. Gradient: 5% to 95% B (0 - 10 min), 95% B (10 – 12 min), 95% to 5% B (12 – 12.1 min), 5% B (12.1 – 15 min). ^{1}HNMR (400 MHz) and ^{13}CNMR (101 MHz) were analyzed with Bruker AV-400MHz Ultra Shield NMR. Data for ^{1}HNMR and ^{13}C NMR are reported in terms of chemical shift (δ, ppm). Multiplicity (s = singlet, d =doublet, t = triplet, q = quartet, m = multiplet or unresolved, br = broad singlet, coupling constant(s) in Hz.). Fluorescence spectrum was recorded on Shimadzu RF-5301PC fluorospectrometer (Kyoto, Japan). Gas chromatography (GC) was analyzed using Agilent 7820A system equipped with purged packed inlet (operates at 150℃), Restek 5A mole sieve column (2m, 0.53mm ID, helium carrier gas at 4.5 mL/min flow rate), Restek CH4izer methanizer coupled with FID detector (methanizer H_2 flow rate 25 ml/min, FID H_2 flow rate 15 mL/min, air flow rate 400 mL/min, methanizer temperature 380 ℃, FID temperature 300 ℃), oven temperature program: 0-4 min 100 ℃ then increase to 250 ℃ at a rate of 60 ℃/min and hold at 250 ℃ for 4 min followed by decrease to 100 ℃ at a rate of 60 ℃/min then hold at 100 ℃ for 2 min. Typical CO peak is eluted at 1.3-1.4 min.

Biology*:* Raw 264.7 cells and H9c2 cells were purchased from the American Type Culture Collection (ATCC, Manassas, VA, USA). Raw 264.7 and H9c2 cells were cultured in Dulbecco's modified Eagle's medium (DMEM, Corning) supplemented with 10% fetus bovine serum (FBS, Corning) and 1% penicillin/streptomycin (PNS). Fetal bovine serum (FBS), Dulbecco's modified Eagle's medium (DMEM) and Trypsin-EDTA (0.05%) were purchased from Gibco BRL (Gaithersberg, Maryland, USA). CO gas treatment was conducted under 37℃ with a gas-tight chamber (2500 mL volume, Mitsubishi Gas Chemical, Japan) equipped with needle valve gas inlet and outlet ports under constant supplement (5 mL/min) of premixed CO gas (250 ppm CO, 5% CO_2 in balanced air, Airgas, PA, USA). Cell incubator: VWR symphony (Radnor, Pennsylvania, USA). Cell counting kit-8 (CCK-8) was purchased from Dojindo (Kumamoto, Japan) and used according to the manufacture's manual. Optical density and microplate fluorescence assay were measured with multi-wavelength plate reader: Perkin-Elmer Vector3 (Shelton, Connecticut, USA). For CCK assay, optical density (OD) was measured at 450 nm; for fluorescence reading, fluorescence intensity (counts per second, CPS) was measured with the excitation filter wavelength of 405 nm and excitation filter wavelength of 535 nm, bandwidth=10 nm, aperture: normal, signal collection time: 2 s per well. Cell imaging was conducted on the Olympus IX73 inverted fluorescence microscope (Tokyo, Japan).

Statistical Analysis: All data were presented as the mean ± standard deviation (n ≥ 3). Statistical analysis was performed by Student's t-test for comparison between two groups or by One-way ANOVA for comparison among multiple groups using GraphPad Prism 9 (San Diego, CA, USA). A *p*-value of less than 0.05 was considered to be statistically significant. Significance level is noted in the figure legend.

Data plotting and figure sketching: Fluorescence spectrum and experimental data were plotted with GraphPad Prism 9 (San Diego, CA, USA). Illustrative figures were created with BioRender.com.

2.2 LCMS Study of QCO-103 under Controlled Oxygen Enviroment

QCO-103 was weighed in a 1.5 mL HPLC vial and was purged with nitrogen gas and was protected by a nitrogen ballon. DMA (500 µL) was purged by bubbling with nitrogen and then was injected into the reaction vessel. Reaction was incubated at 37 °C and monitored with HPLC by repeated injection from the same vial every 30 min.

2.3 GC Studies of QCO-103 and QCO-105 under Controlled Oxygen Environment Using Two-compartment Setup. **QCO-103** or **QCO-105** was weighed in to an 250 µL HPLC vial insert and placed in the headspace vial (8.8 mL inner volume) as shown in the below pictures. The nitrogen purged solvent (DMA or pH 5.8/DMSO (1:9) mixture was added to the vial in the periphery of the insert. The vial was sealed by a crimping silicon rubber septum with PTFE membrane. After purging the whole headspace vial (including the solvent) with nitrogen via a needle for 20 min. The solid was then dissolved in the surrounding solvent mixture by inverting and shaking the vial followed by incubation for 30 min. Then 100 µL of the headspace gas was taken out by GC syringe and injected to GC to analyze CO content.

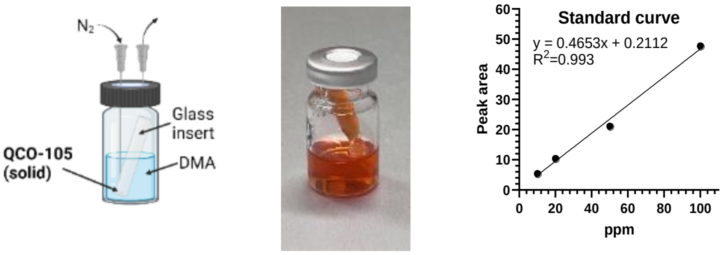

A two-compartment setup for oxygen-free experiment (left) and the actural picture of reaction of **QCO-105** (middle). GC standard curve for quantification (right)

2.4 Electrochemical Measurements and Data Analysis

Voltammetric measurements were performed using a CHI 750C electrochemical workstation (CH Instruments, Inc., Austin, TX). Cyclic voltammograms (CV) and squre wave voltammograms (SWV) were recorded with a 3 mm diameter glassy carbon (GC) working electrode against a Ag/Ag^+ reference electrode (0.01 M silver nitrate and 0.1 M tetrabytylammonium hexaflurophosphate (TBAPF6) in anhydrous acetonitrile), and a Pt counter electrode. The QCO 105 and QCO 103 were prepared by dissolving in DMSO (Sigma Aldrich) containing 0.1 M tetrabutylammonium perchlorate (TBAP) supporting electrolyteat at about 1.8 mg and 2.2 mg respectively in 7.5 mL solution under Ar atmosphere, and then mixed with Ar purged pH 5.8 PBS solution at 9:1 volume ratio. The sample solution was under continuous Ar purging (gas inlet right above yet without agitating the solution) during electrochemical data collection.

For CV, current was recorded at 1 mV step size while the potential was scanned cyclically at the listed scan rates. For SWV, cathodic and anodic scan was recorded at 10 Hz frequency with 4 mV stepsize and 25 mV amplitude. Formal potentials (E ½) of the oxidation and reduction redox processes were determined from the peak positions in SWVs, and corraborated with the corresonding CV redox peaks in both potential range and current amplitudes. The reading error is estimated to be less than 10 mV.

Mechanistic understanding of the transformation of QCO to QCP compounds is supported by electrochemical analysis summarized in Fig. S10. For **QCO-105**, the peak separation of a prominent redox process (- 0.55_6 V minus - 0.49_7 V) is about 59 mV, the signature of electrochemical reversibility which is independent of scan rates. The formal potential ($E_{1/2}$) is determined to be - 0.51_2 V from the square wave voltammetry (SWV) peaks. Another reversible redox process can be seen with a formal potential of - 0.90_8 V (Fig. S10 D/E). With comparable cathodic current amplitudes, the two peaks are attributed to the sequential 1e reduction of quinones.[1, 2] The $E_{1/2}$ is at – 0.31_4 V for the first reversible redox process of **QCO-103** (- 0.41_8 V minus - 0.36_2 V). However, the second redox process with the $E_{1/2}$ of – 0.7_4 V becomes more obvious only at higher scan rates. Interestingly, the – 0.31_4 V peak diminishes over time and the – 0.7_4 V becomes the first prominent reduction process, associated with the introduction of O_2 (i.e., open to the air without Ar purging) into the measurement solution and/or complete CO release (Fig. S10 B/C). A chemically irreversible anodic peak, of the enol form, is observed at + 0.15_3 V for **QCO-103** and + 0.28_8 V for **QCO-105**. Importantly, the two reversible reductions between 0 to – 1V are only detected after this oxidation process. Such observations under different scan rates are consistent with the fast

cheletropic reaction which may lead to the consumption of the reducible intermediate species (*e.g.* **IM-3** or **IM-5**) on the electrode under a slower scan rate. The electrode oxidation-initiated redox features confirm the essential nature of the oxidation step for CO release, as proposed. Further, the more positive oxidation potential (+ 0.28_8 V vs + 0.15_3 V; or more negative in reduction half-cell reaction format) explains the more complete or spontaneous reaction of **QCO-105** over **QCO-103** with O_2, consistent with the observation that the solution turns dark red from light yellow more promptly for **QCO-105**. Other irreversible reductions at more negative potentials are presumably associated with original tautomerization mixtures.

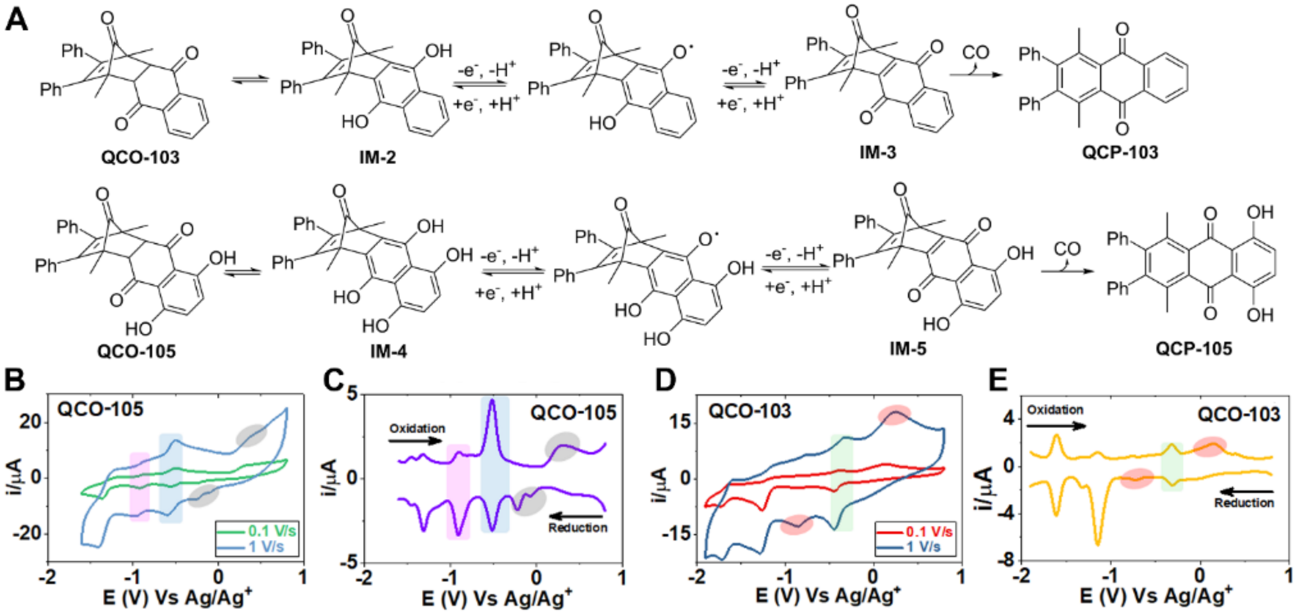

Figure S10. **Cyclovoltammetric studies of QCO-103 and QCO-105**. (A) Proposed redox reaction mechanism; (B) cyclovoltammetry of **QCO-105**; (C) SWV of **QCO-105;** (D) cyclovoltammetry of **QCO-103**; (E) SWV of **QCO-103;** Color boxes indicate the redox peaks discussed in the text. Peak positions are listed in SI (Table S1).

Table S1. Cyclic voltammetry and squarewave voltammetry data associated with Figure S10.

**Cyclic voltammetry**	**QCO-103**		**QCO-105**	
E_{Ox} (V)	-0.79_7	-0.36_2	-0.86_3	-0.49_7
E_{Red} (V)	-0.83_2	-0.41_8	-0.91_3	-0.55_6
**SWV**	**QCO-103**		**QCO-105**	
Irreversible (V)	0.153		0.288	
$E_{1/2}$ (V)	-0.74	-0.314	-0.908	-0.512
Color code	red	green	magenta	blue

2.5 ELISA Assay for TNF-α Level in RAW264.7 Cell Culture

Anti-inflammatory effects of **QCO-103** and **QCO-105** were tested in RAW246.7 cells. Cells were cultured in 96-well plate at a density of 1×10^4/100 μL/well with DMEM medium supplemented with 10% fetus bovine serum and 1% penicillin and streptomycin. After culturing overnight to allow cell adhesion, **QCO/QCP-103** and **QCO/QCP-105** were made into stock solution using dry DMSO and quickly made into cell culture working solution by serial dilution with full DMEM culture medium. DMSO concentration was kept equal as 0.5%. Cells were incubated with the compound at 37 °C for 5 h, then 10 μL of 10 μg/mL of LPS in PBS was added to the cell culture medium and incubated for 1 h. The control group was added with 10 μL PBS. The supernatant culture medium was diluted 5× by ELISA assay diluent and TNF-α concentration was then determined with ELISA kit (Biolegend, USA) following the manufacturer's protocol. The standard curve of the standard sample is shown in the figure below.

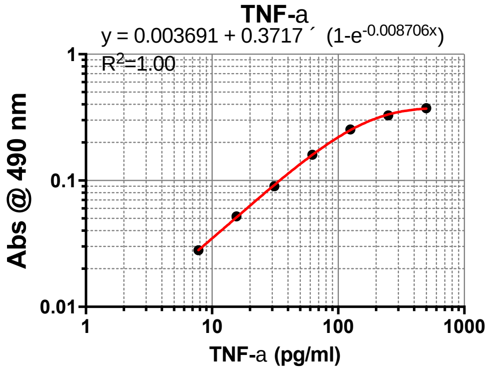

Standard curve for quantification of TNF-α in the RAW264.7 cell culture.

2.6 Cell Imaging with CODP-202 and COP-1

RAW264.7 cells were seeded in the 3.5 cm Cellvis glass bottom culture dish (Mountain View, California, USA) at a density of 1×10^3/dish in FluoroBrite DMEM medium (ThermoFisher, Carlsbad, California, USA) supplemented with 10% FBS, 100 units penicillin, and 100 μg/mL streptomycin. After culturing overnight, the cells were treated with designated conditions and incubated for 1 h: vehicle control: 0.5% DMSO in culture medium, 50 μM **QCO-103**. Then CODP-202 or COP-1 was added to the culture medium at a concentration of 10 μM or 5 μM, respectively. CODP-202[3] and COP-1[4] were synthesized according to the reported procedures. Cells were continuously incubated under the previous conditions for 1 h. Cells were then washed twice with PBS and incubated in fresh FluoroBrite DMEM medium. The live cells were imaged with Olympus IX-73 inverted fluorescence microscope under DAPI channel (for CODP-202) or FITC channel (for COP-1) and phase-contrast transillumination settings.

2.7 Western-blot of HO-1 in RAW264.7 Cells

Primary monoclonal antibodies (mouse origin) for HO-1 (sc-390991) and beta-actin (sc-8432) were purchased from Santa Cruz Biotechnology (Texas, USA). The secondary antibody (goat-anti-mouse HRP conjugated IgG) was purchased from Bio-rad (Hercules, California, USA). Cells were seeded in a 6-well plate and treated after confluency reached about 80-100%. After treating the cells with specified conditions: for RAW264.7 cells, cells were incubated with various concentrations of **QCO-/QCP-103** and **QCO-/QCP-105** as indicated in the manuscript; another 6-well plate containing RAW264.7 cells with the same culture density were incubated in the CO chamber with premixed 250 ppm CO gas placed in the 37°C cell incubator for designated time. For H9c2 cells, cells were seeded in two 6-well plates. After reaching 80% confluency, the culture medium was replaced with the medium containing 50-200 μM hemin or 0.1-0.2 μM CDDO-Me. Culture medium containing 0.5% DMSO was used as the vehicle control. One 6-well plate was placed in a normal cell incubator with 5% CO_2 at 37°C while the other plate was placed in CO gas chamber containing 250 ppm CO gas and 5% CO_2 in balanced air at 37°C. After treatment, culture medium was aspirated, and the cells were washed three times with PBS. Cells were lysed with 120 μL 1× Laemmli loading buffer (contains 2.5% mercaptoethanol) and denatured at 95°C for 5 min. 2-5 μL denatured cell lysate was loaded onto the 10% SDS-PAGE gel (Bio-rad) and electrophoresis was conducted with Bio-rad system under constant voltage (150 V). The protein was transferred to the PVDF membrane with the Trans-Blot Turbo RTA transfer kit (Bio-rad). The membrane was blotted by the iBind Flex system (ThermoFisher), using HO-1 (1:100), beta-actin (1:400), and HRP-conjugated secondary antibody (1:1500). The chemiluminescent of the protein band was imaged with LAS4000 (GE Healthcare, Chicago, IL, USA).

2.8 Cytotoxicity Assay to Assess the Protective Activity of QCO-105 against Doxorubicin-induced Toxicity

H9c2 cell line was cultured in DMEM culture medium containing 10% FBS, 1% PNS, in humidified air with 5% CO_2 at 37 °C. After reaching 90% confluency, cells were trypsinized and seeded onto a 96-well plate (Corning, Corning, MA, USA) at a density of 1×10^4 cells/100 μL/well and cultured until reaching full confluency. The cells were treated with different concentrations of **QCO-105** and **QCP-105.** DMSO (0.5%) was used as the vehicle control. After incubating for 3 h in the cell incubator, the cell culture medium was carefully removed and the fresh full DMEM culture medium containing

5 µM doxorubicin was added to the cell culture wells. 0.5% DMSO treated cells (without DXR and QCO/QCP treatments) were used as the negative control group. After incubating for 24 h in the cell incubator, the culture medium was carefully replaced with fresh full culture medium containing 10% Cell Counting Kit-8 (CCK-8, Dojindo, Kumamoto, Japan) and incubated for another 2 h. The cell density of each well was determined by the absorption at 450 nm with multiplate reader (Perkin-Elmer Vector-3, MA, USA). The wells containing only CCK-8 culture medium without the cells were used as the blank control wells. Each treatment condition was triplicated (n = 3). Cell availability was calculated according to the formula:

$$Cell\ availablity\ (\%) = \frac{Abs_{treatment\ well} - Abs_{blank\ well}}{Abs_{negative\ control} - Abs_{blank\ well}} \times 100\%$$

2.9 QCO-105 Treatment for Doxorubicin-Induced Cardiac Dysfunction

Ten-to-12-week-old male C57BL/6 mice (Jackson Laboratories) were housed under specific pathogen-free conditions with 12-hour day/light cycles. All mouse procedures were approved by the Beth Israel Deaconess Medical Center, Boston, MA, Institutional Animal Care and Use Committee (#083-2021).

2.9.1 Doxorubicin Treatment Regimen

DXR HCl (D1515, Sigma-Aldrich) regimens were designed to mimic that used in humans, with a recommended therapeutic dose of 60 to 75 mg/m^2 every 21 days in cancer patients.[5] The **QCO-105** and **QCP-105** were tested in combination with DXR. One hour after dosing with **QCO** or **QCP-105** (50 mg/kg, p.o., formulated with solutol**)**, or solutol control, animals were infused with a single dose of DXR (20 mg/kg, i.p.; ~60 mg/m^2; 3-7 mice per group). We tested an acute toxicity regimen with hearts harvested 7 days after DXR administration. The presence of DXR-induced cardiomyopathy was confirmed by mRNA quantification of the cardiac dysfunction markers (B-type Natriuretic Peptide, BNP: Nppb; and beta Myosin Heavy Chain, β-MyHC: Myh7) and measurement of cardiac function by echocardiography compared with control solutol-treated mice (see 2.8.2). The ventricles were carefully collected, frozen in liquid nitrogen, and stored at −80 °C until analysis.

2.9.2 Echocardiography

Heart structure and function assessments were conducted using echocardiography (Vevo 770, Visual Sonics, Toronto, Canada) with animals placed under isoflurane anesthesia (2% v/v). Animals in the acute DXR regimen underwent pre- and post-experimental protocol echocardiography sessions. Fractional shortening (FS) was calculated using LVIDd and LVIDs as follows: FS (%) = [(LVIDd - LVIDs)/LVIDd] x 100. Moreover, ejection fraction percentage (EF %) was assessed. All measurements of myocardial structures were manually performed following the leading-edge method of the American Society of Echocardiography.[6] The examiner (RWAS) was blinded to the group allocations.

2.9.3 RT-qPCR

Total RNA was extracted from heart ventricles using TRizol reagent (Invitrogen) following the manufacturer's instruction. Reverse transcription was performed using a High-Capacity cDNA synthesis kit (Thermo Scientific). After cDNA synthesis, RT-qPCR for target and endogenous genes (Table S2) ran separately, and amplifications were detected using QuantStudio3 (Applied Biosystems, Foster City, CA, USA) using Maxima SYBR Green/ROX qPCR Master Mix (Thermo Scientific). Fold-change was calculated through the ΔΔCt method.

Table S2. Primer sequences for RT-qPCR mRNA analysis.

Target	PCR Primer sequence 5′ → 3′	Product Size (bp)	GenBank Accession #
Hmox1	F: CAGAAGAGGCTAAGACCGCC<br>R: AGCTCCTCAAACAGCTCAATGT	52	NM_010442
Nppb	F: TTTGGGCTGTAACGCACTGA<br>R: CACTTCAAAGGTGGTCCCAGA	113	NM_001287348
Myh7	F: CCTGCTGTTTCCTTACTTGCT	84	NM_080728

	R: CCAGGCCTGTAGAAGAGCTGTA		
Gpx4	F: CATTGGTCGGCTGCGTGAG<br>R: TTAAGTAAGCGGCTCAGACGG	80	NM_008162
Hprt1	F: CAGTCCCAGCGTCGTGATT<br>R: GCAAGTCTTTCAGTCCTGTCCAT	138	NM_13556

bp indicates base pairs; RT-qPCR, reverse transcription quantitative polymerase chain reaction.

3. NMR spectrum

QCO-101

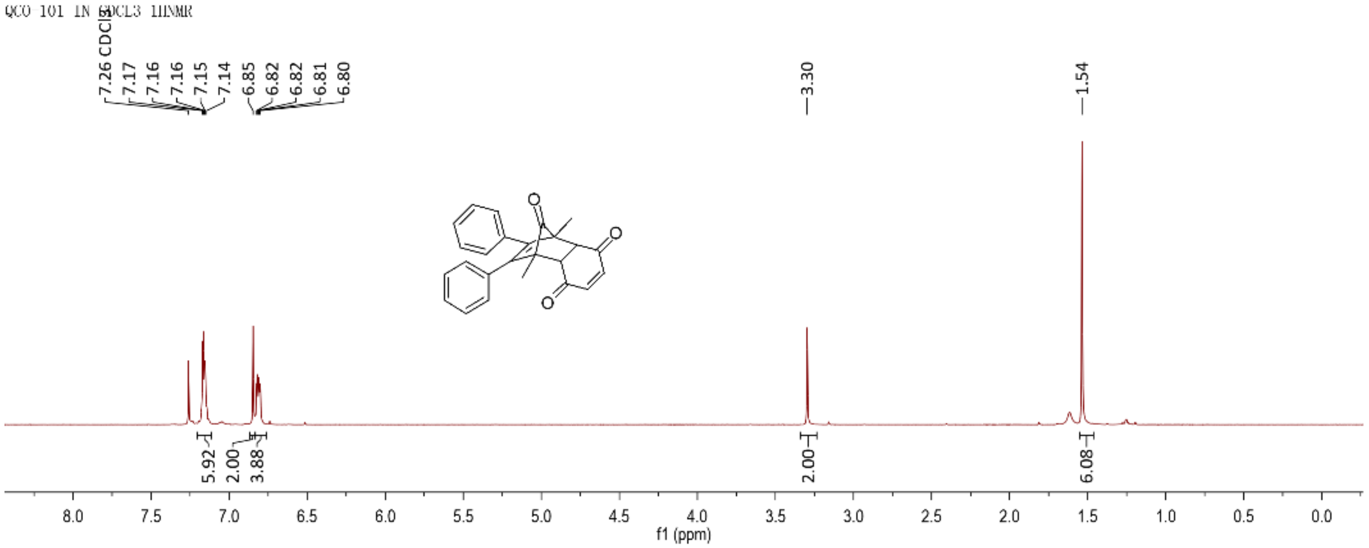

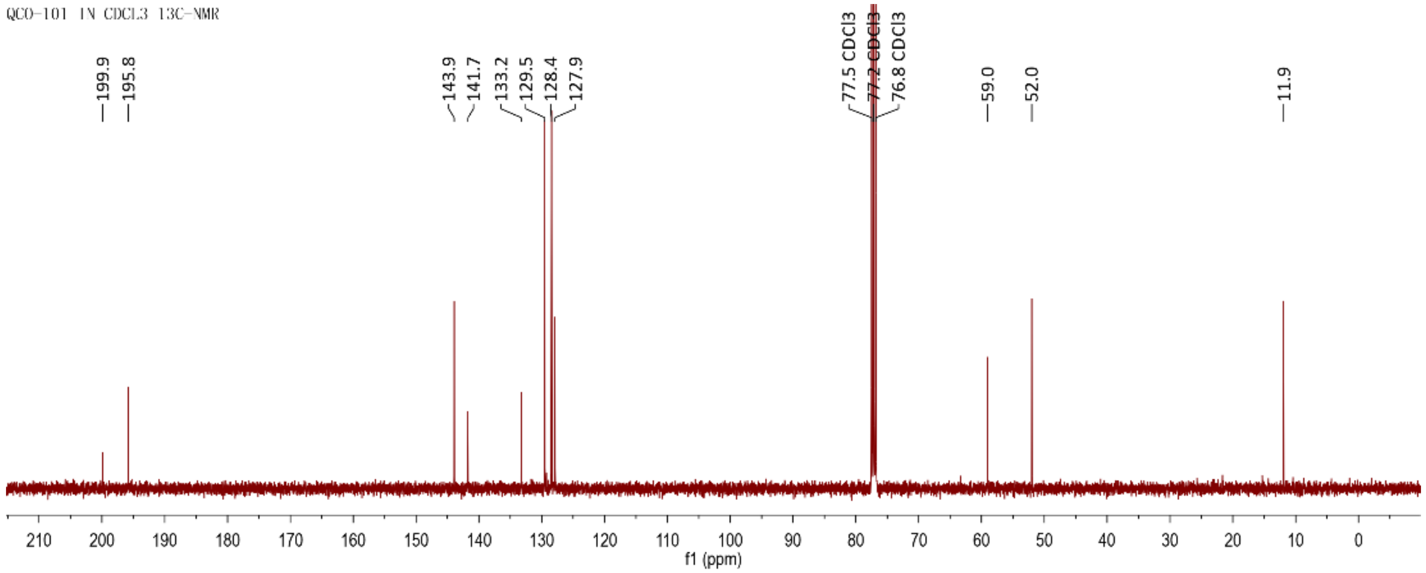

IM-1

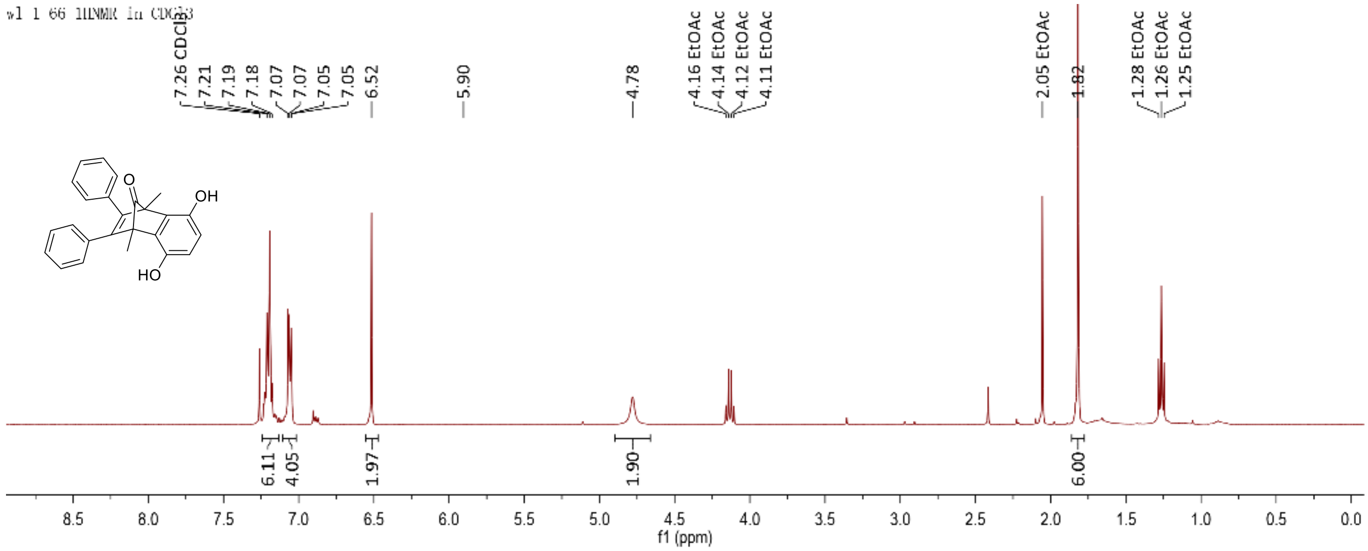

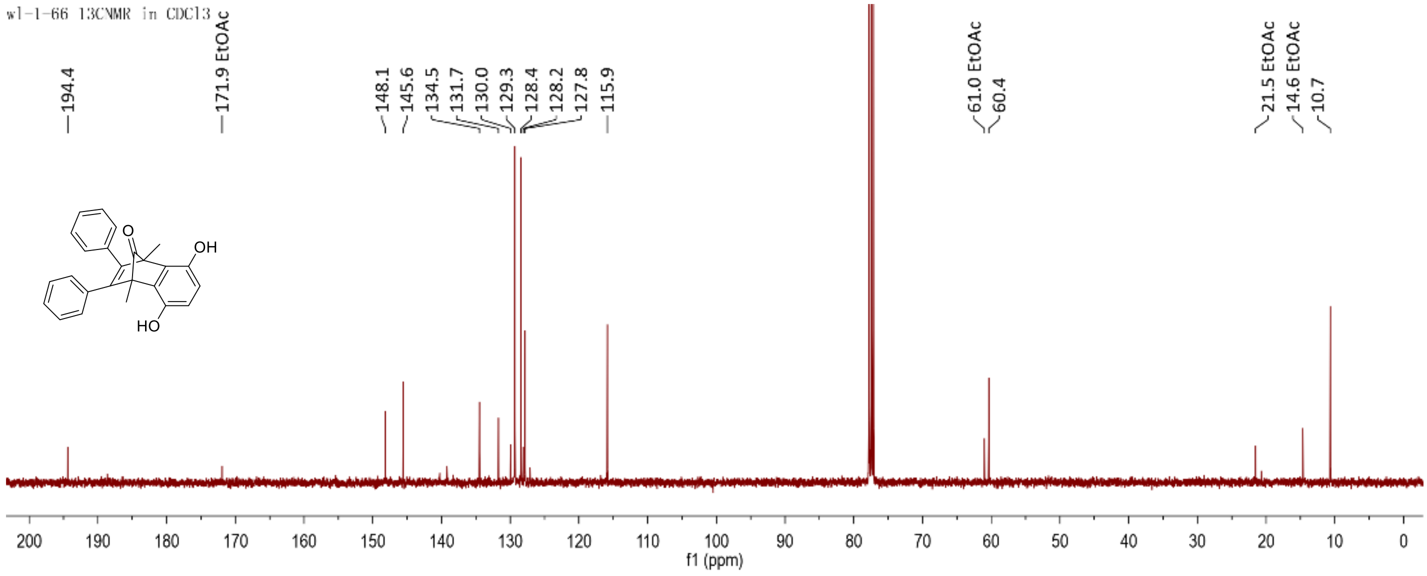
wl-1-66 13CNMR in CDCl3
194.4
171.9 EtOAc
148.1
145.6
134.5
131.7
130.0
129.3
128.4
128.2
127.8
115.9
61.0 EtOAc
60.4
21.5 EtOAc
14.6 EtOAc
10.7
O
OH
HO
f1 (ppm)

QCO-103

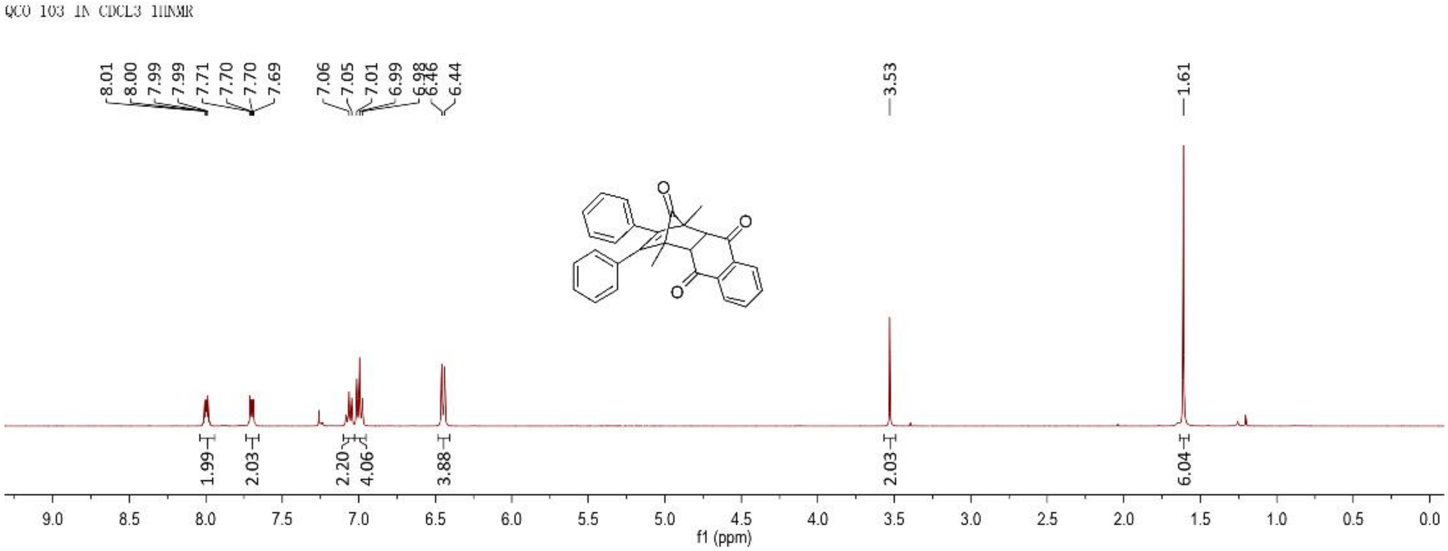
QCO 103 IN CDCL3 1HNMR
8.01
8.00
7.99
7.99
7.71
7.70
7.70
7.69
7.06
7.05
7.01
6.99
6.98
6.46
6.44
3.53
1.61
O
O
O
1.99
2.03
2.20
4.06
3.88
2.03
6.04
f1 (ppm)

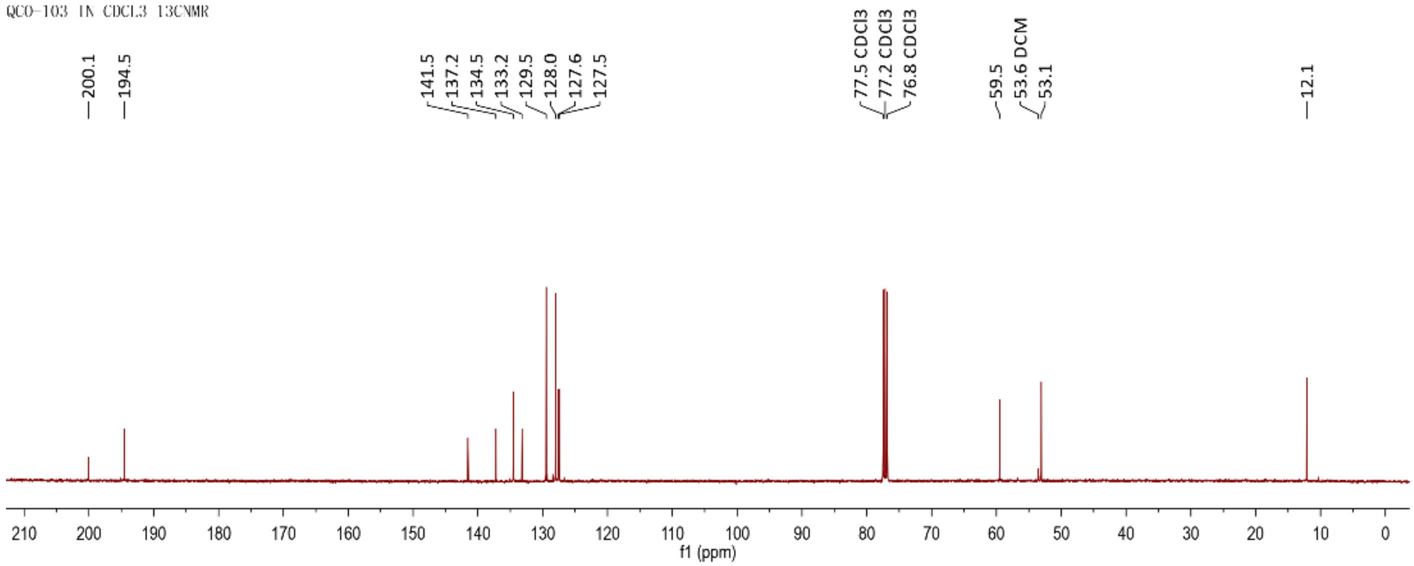
QCO-103 IN CDCL3 13CNMR
200.1
194.5
141.5
137.2
134.5
133.2
129.5
128.0
127.6
127.5
77.5 CDCl3
77.2 CDCl3
76.8 CDCl3
59.5
53.6 DCM
53.1
12.1
f1 (ppm)

QCO-105

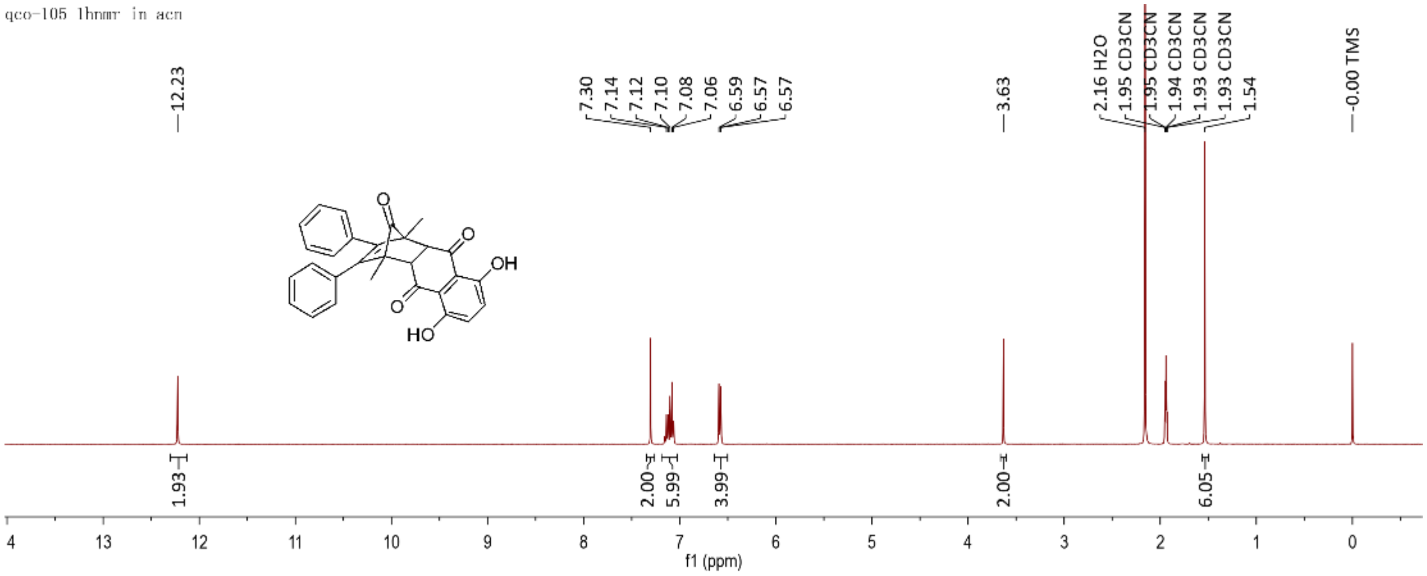

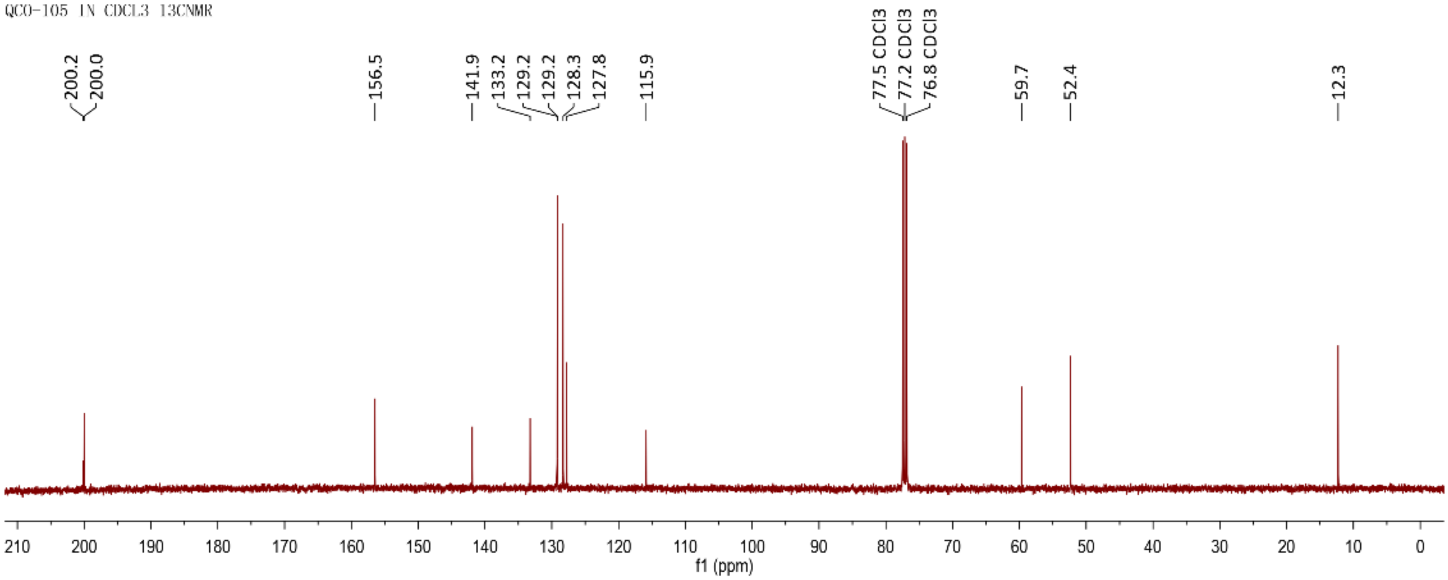

QCP-101

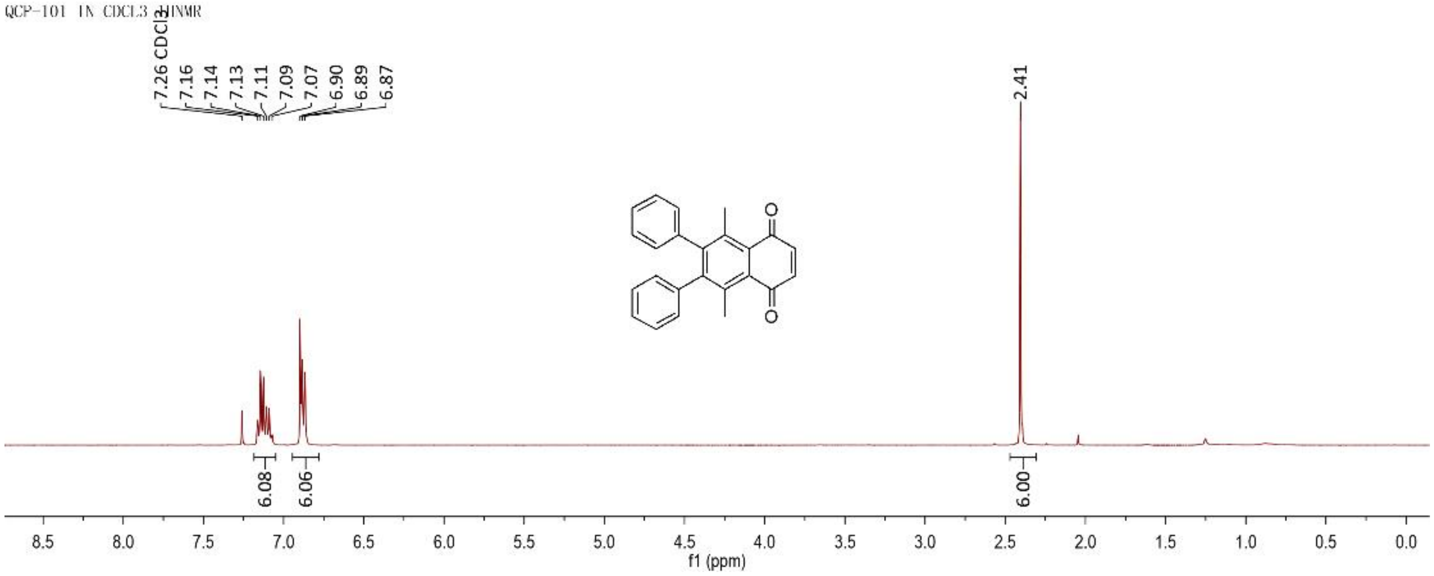

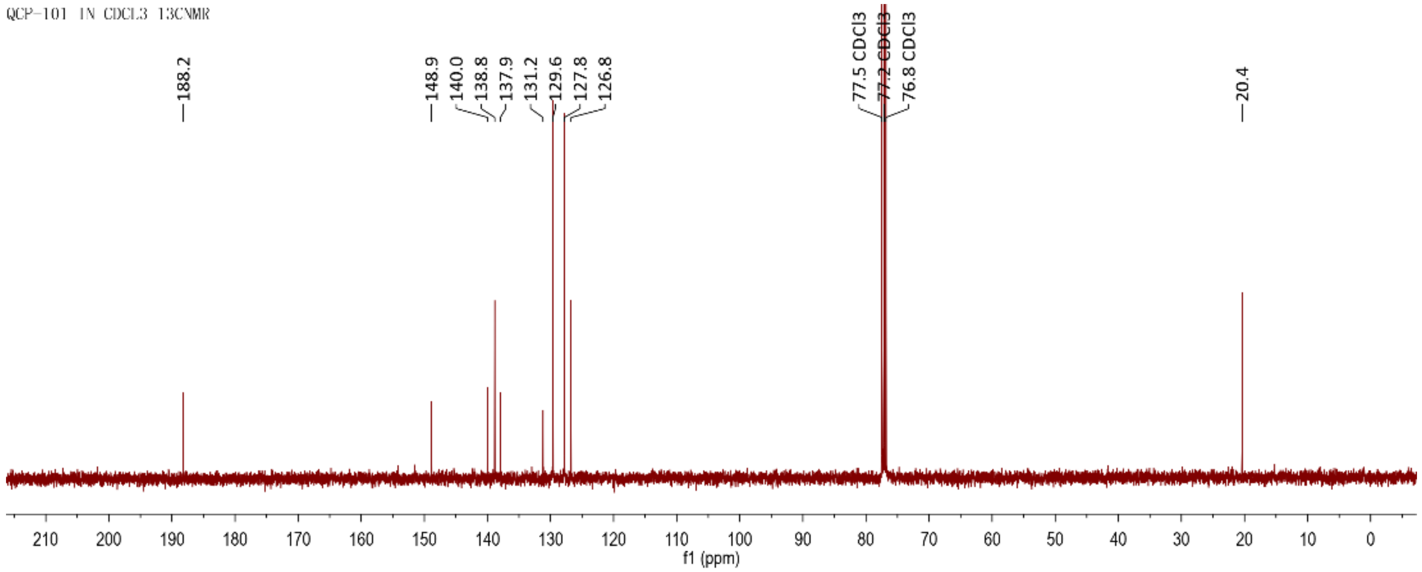
QCP-101 IN CDCL3 13CNMR
188.2
148.9
140.0
138.8
137.9
131.2
129.6
127.8
126.8
77.5 CDCl3
77.2 CDCl3
76.8 CDCl3
20.4
f1 (ppm)

QCP-103

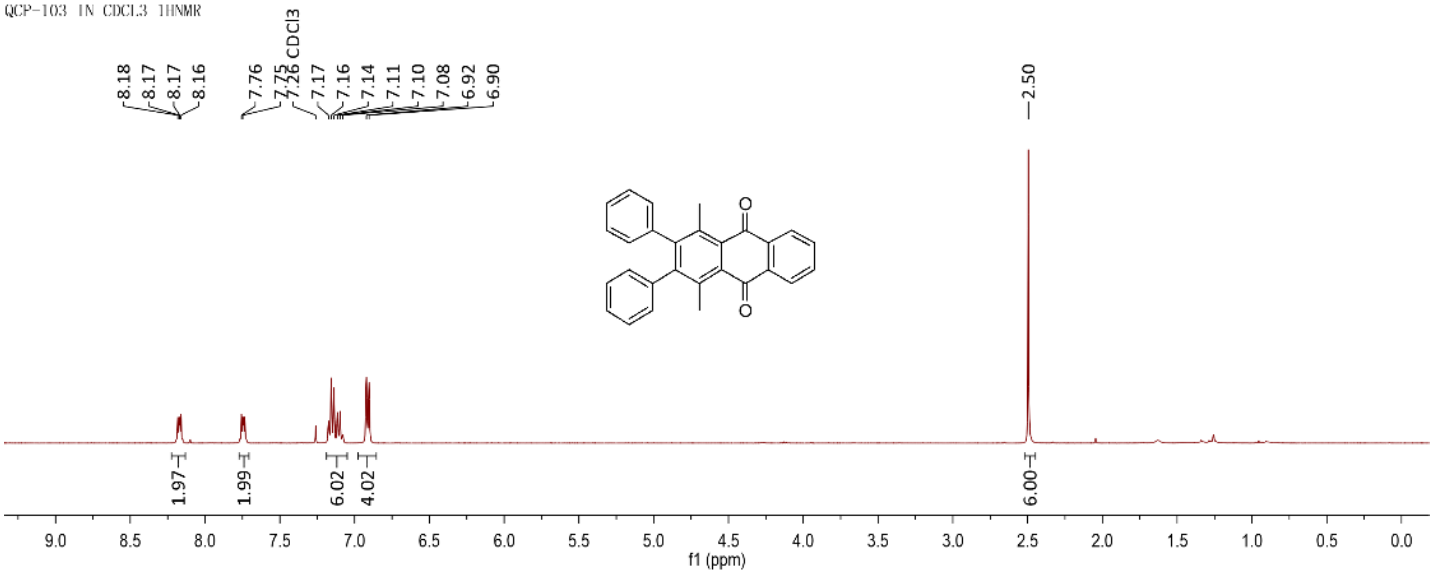
QCP-103 IN CDCL3 1HNMR
8.18
8.17
8.17
8.16
7.76
7.75
7.26 CDCl3
7.17
7.16
7.14
7.11
7.10
7.08
6.92
6.90
2.50
1.97
1.99
6.02
4.02
6.00
f1 (ppm)

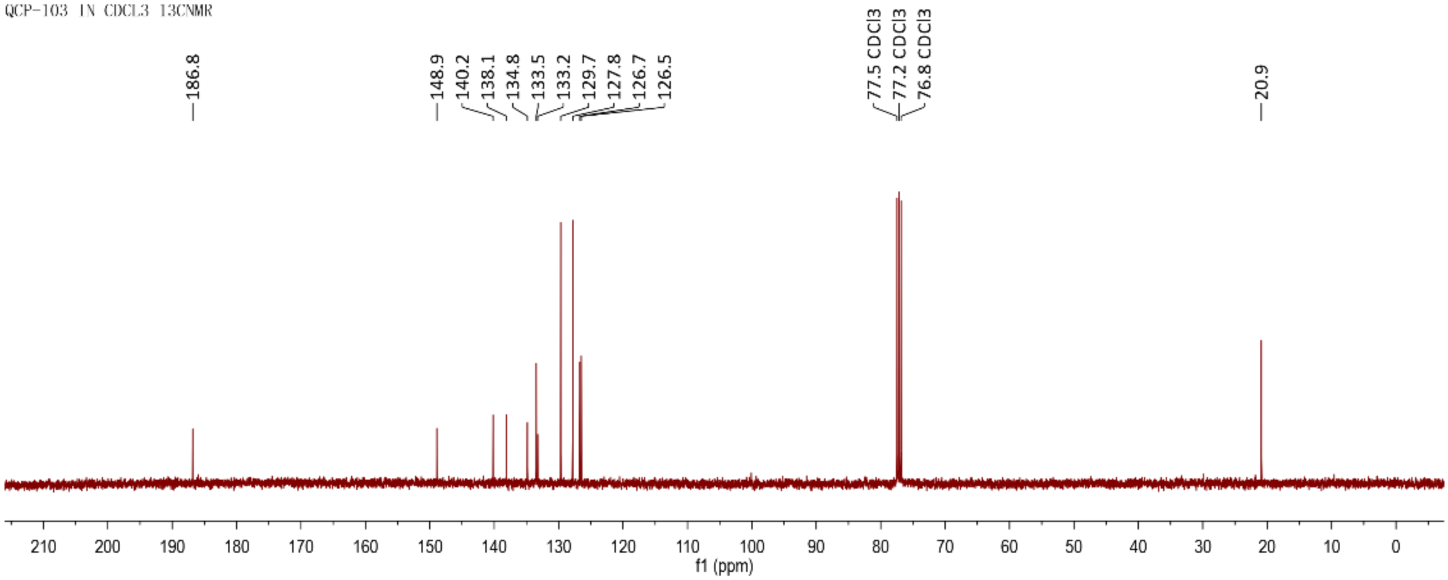
QCP-103 IN CDCL3 13CNMR
186.8
148.9
140.2
138.1
134.8
133.5
133.2
129.7
127.8
126.7
126.5
77.5 CDCl3
77.2 CDCl3
76.8 CDCl3
20.9
f1 (ppm)

QCP-105

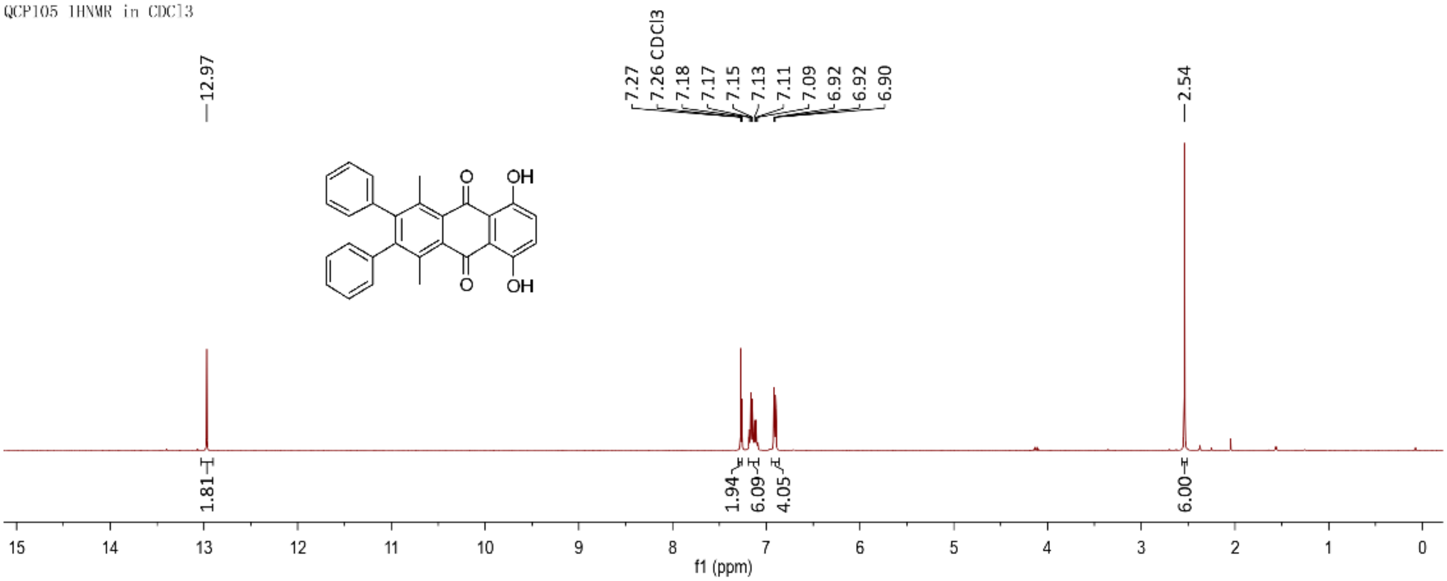
QCP105 1HNMR in CDCl3
12.97
7.27
7.26 CDCl3
7.18
7.17
7.15
7.13
7.11
7.09
6.92
6.92
6.90
2.54
O
OH
O
OH
1.81
1.94
6.09
4.05
6.00
f1 (ppm)

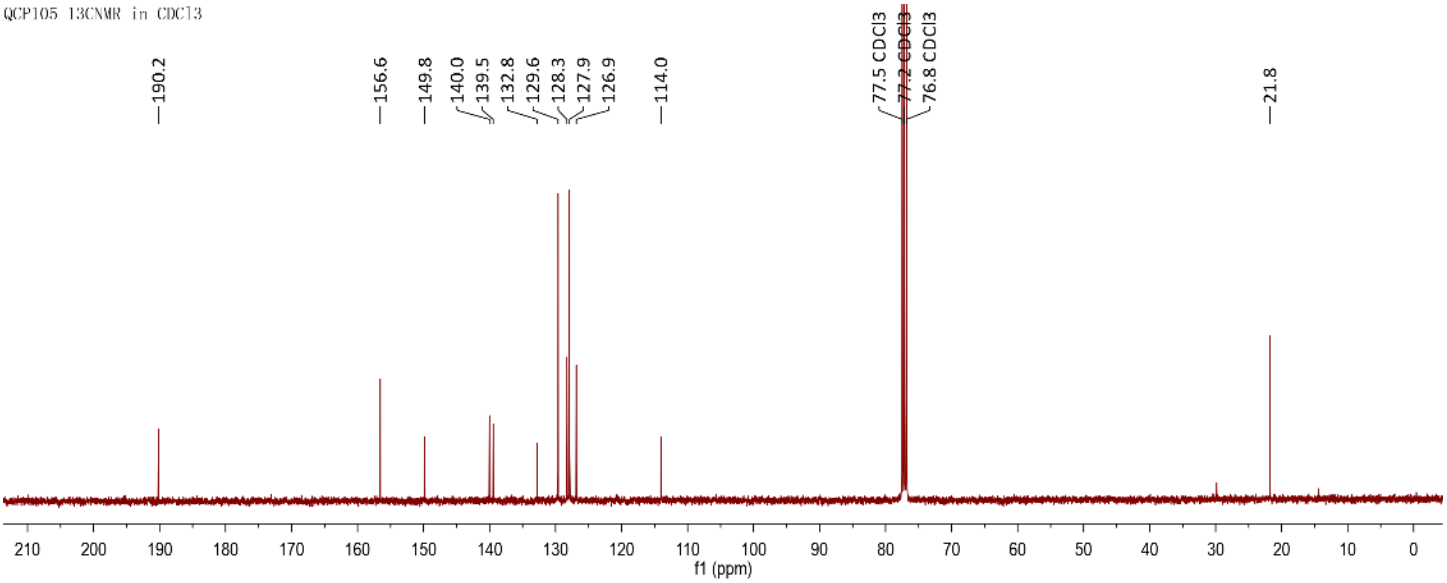
QCP105 13CNMR in CDCl3
190.2
156.6
149.8
140.0
139.5
132.8
129.6
128.3
127.9
126.9
114.0
77.5 CDCl3
77.2 CDCl3
76.8 CDCl3
21.8
f1 (ppm)

5. LC-MS chromatograph of QCO-105 reaction mixture

QCO-105 (m/z=449.0, [M-H]⁻) in DMSO before addition of phosphate buffer:

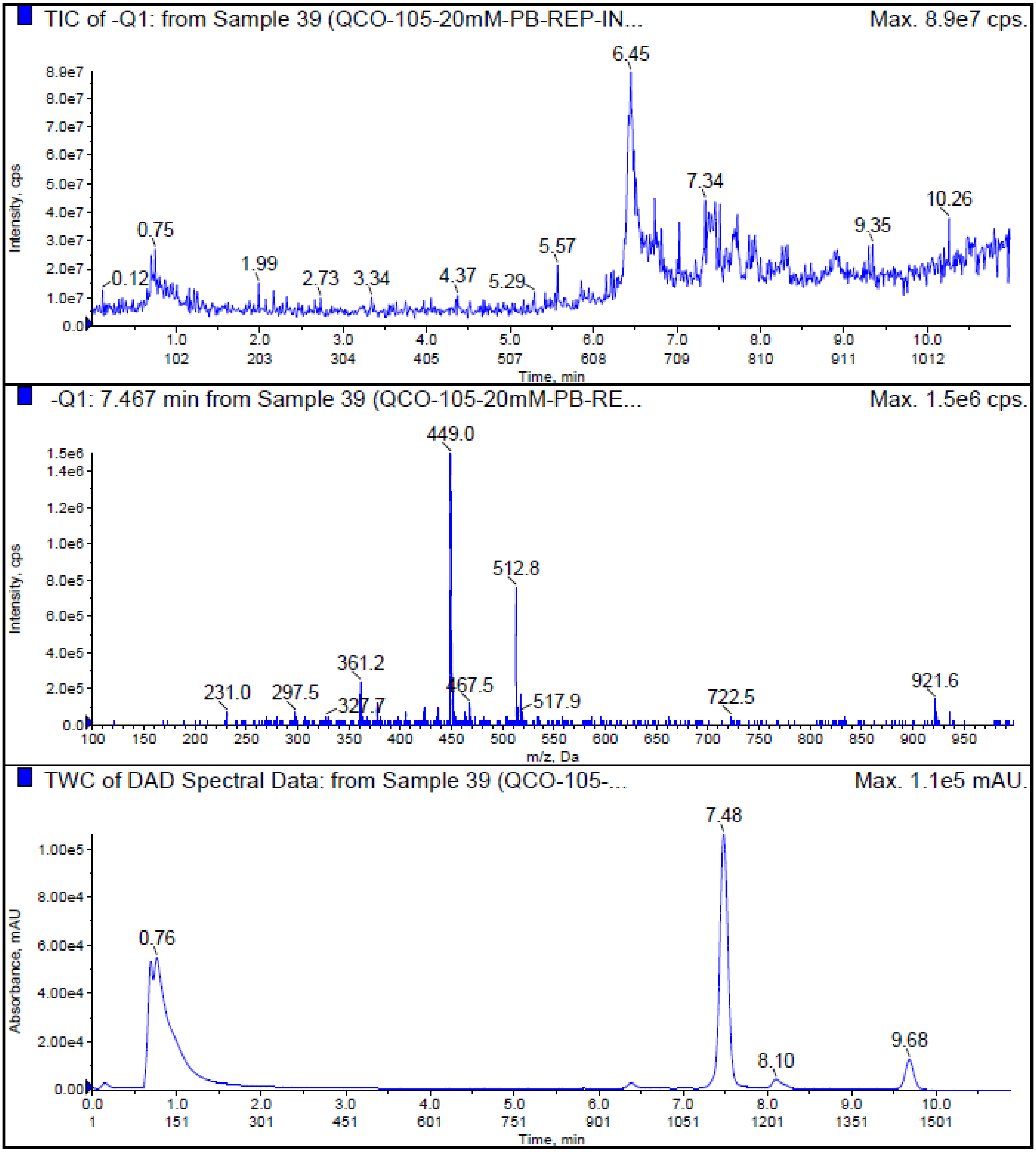

QCP-105 (m/z=421.0, $[M+H]^+$) formed by incubating QCO-105 in DMSO/PB buffer (20 mM) at 20 ℃ for 1 h:

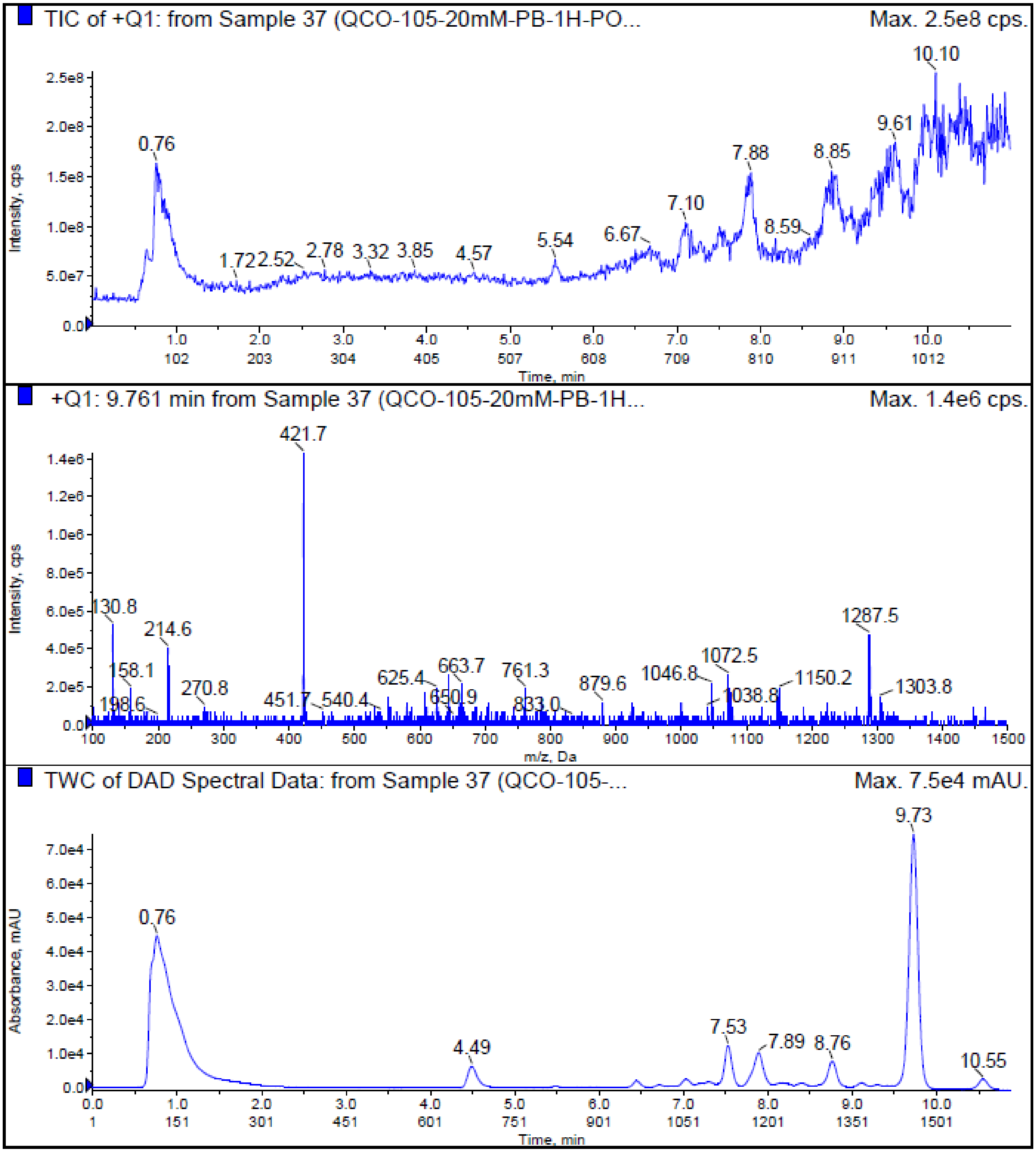

Enol intermediate IM-3 (m/z=449, $[M-H]^-$) formed by incubating QCO-105 in DMSO/PB buffer (20 mM) at 20 ℃ for 15 min:

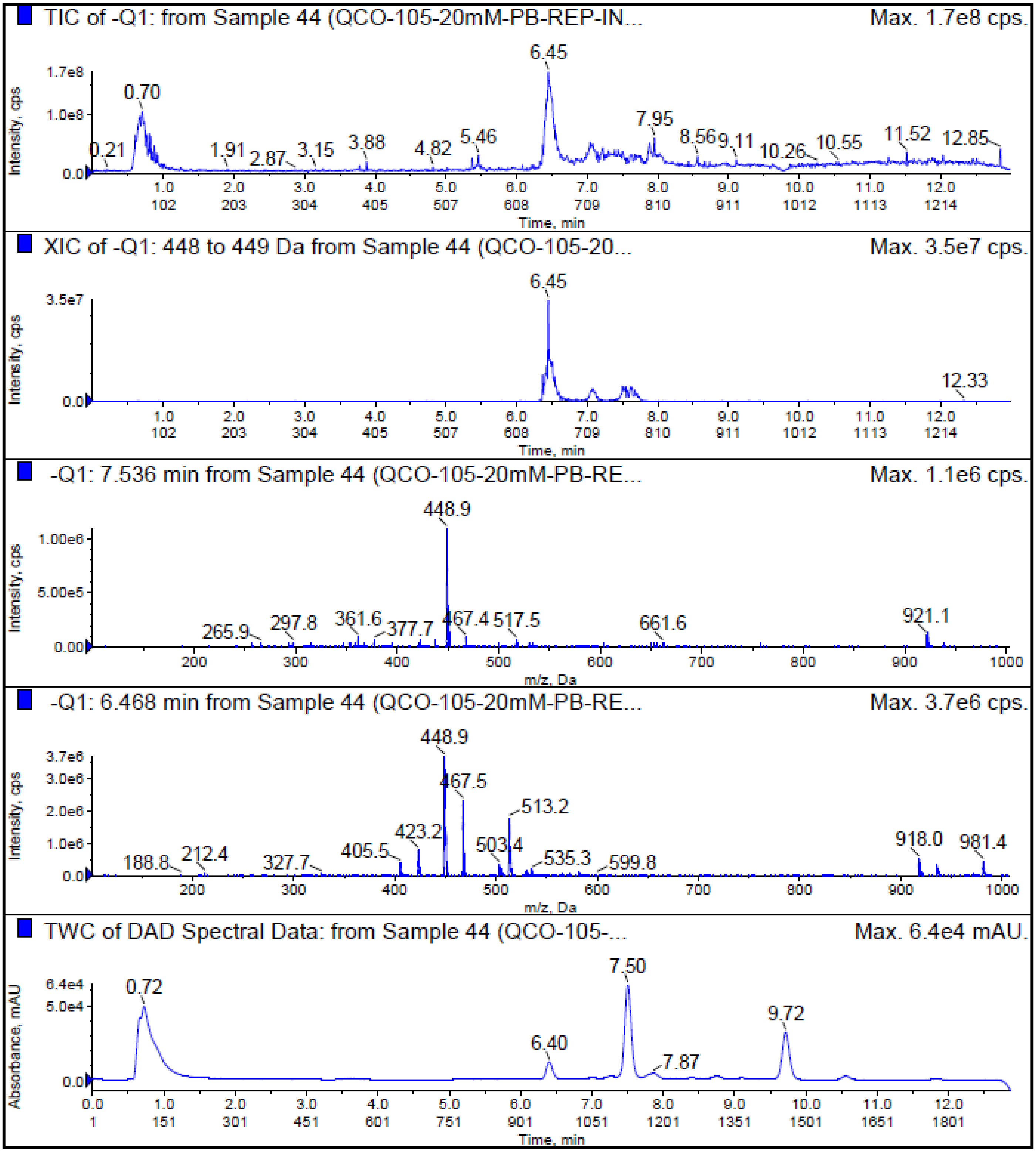

Enol intermediate IM-3 (m/z=449, [M-H]$^-$) formed by incubating QCO-105 in DMSO/PB buffer (5 mM) at 20 ℃ for 15 min:

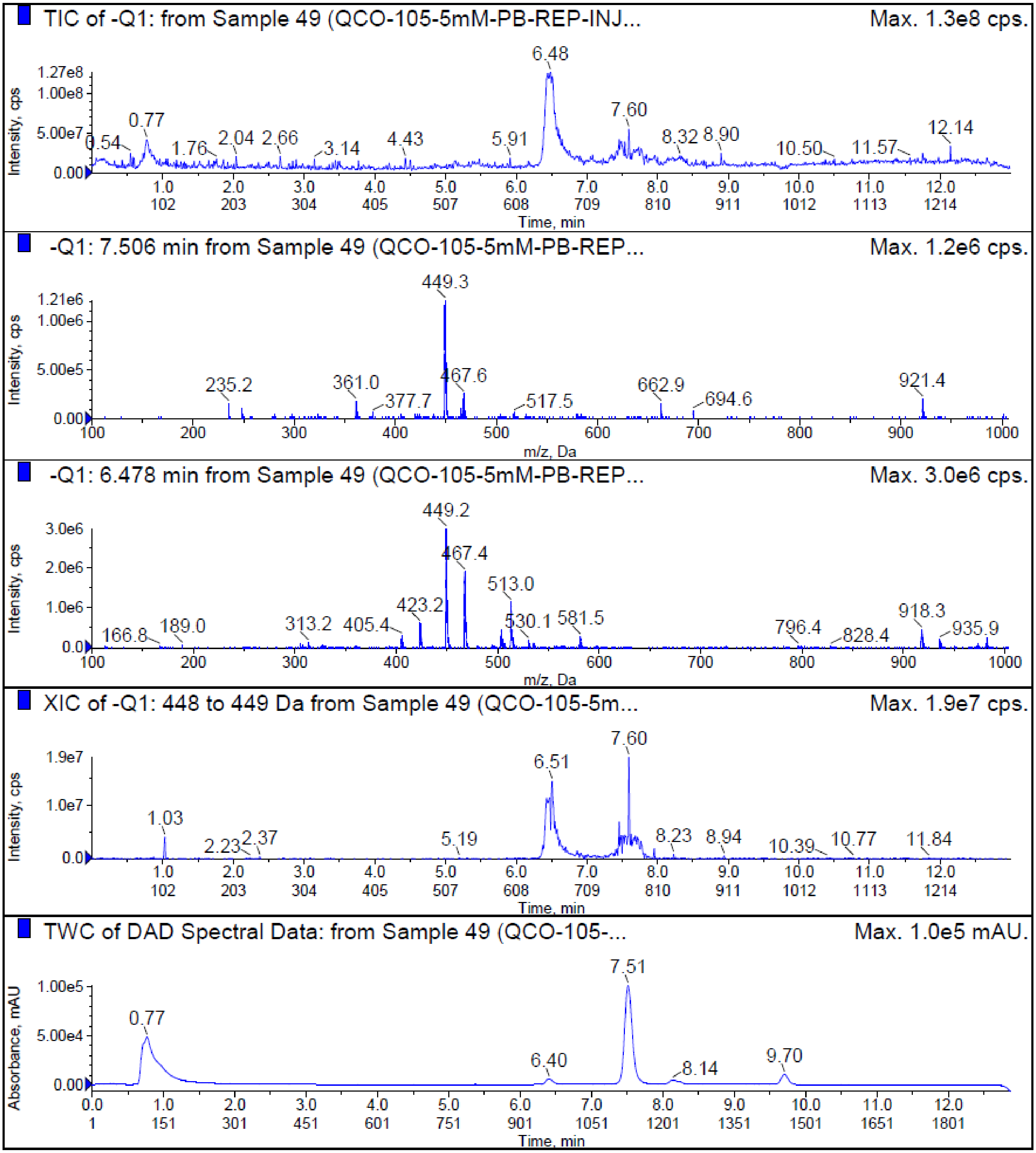
TIC of -Q1: from Sample 49 (QCO-105-5mM-PB-REP-INJ...
Max. 1.3e8 cps.
Intensity, cps
Time, min
6.48
0.77
7.60
0.54
1.76
2.04
2.66
3.14
4.43
5.91
8.32
8.90
10.50
11.57
12.14
-Q1: 7.506 min from Sample 49 (QCO-105-5mM-PB-REP...
Max. 1.2e6 cps.
Intensity, cps
m/z, Da
449.3
235.2
361.0
377.7
467.6
517.5
662.9
694.6
921.4
-Q1: 6.478 min from Sample 49 (QCO-105-5mM-PB-REP...
Max. 3.0e6 cps.
Intensity, cps
m/z, Da
449.2
467.4
513.0
423.2
166.8
189.0
313.2
405.4
530.1
581.5
796.4
828.4
918.3
935.9
XIC of -Q1: 448 to 449 Da from Sample 49 (QCO-105-5m...
Max. 1.9e7 cps.
Intensity, cps
Time, min
7.60
6.51
1.03
2.23
2.37
5.19
8.23
8.94
10.39
10.77
11.84
TWC of DAD Spectral Data: from Sample 49 (QCO-105-...
Max. 1.0e5 mAU.
Absorbance, mAU
Time, min
7.51
0.77
6.40
8.14
9.70

6. Elemental analysis of QCO-105 and HPLC traces of QCP-105 used in the in-vivo animal studies.

(1) QCO-105 Due to the transformation of QCO-105 in the HPLC sample vial and HPLC system it is not feasible to provide HPLC purity of QCO-105. Alternatively, elemental analysis (CHN analysis) was done to verify the purity of QCO-105 that the difference between theoretical calculation and found elemental composition is less than 0.5%.

Sample No. QCO-105

6180 Atlantic Blvd. Suite M
Norcross, GA 30071
www.atlanticmicrolab.com

Company/School Georgia State University
Dept. Chemistry Department
Address 100 Piedmont Ave SE
City, State, Zip Atlanta
Name Xiaoxiao Yang Date 05/16/2024
Phone [redacted]

Professor/Supervisor: Binghe Wang
PO# / CC#

Element	Theory	Found	
C	77.32	77.42	77.45
H	4.92	4.92	4.98

Single ☐ Duplicate ☒
Elements Present: CHO
Analyze for: C29 H22 O5
Hygroscopic ☐ Explosive ☐
M.P. ____ B.P. ____
To be dried: Yes ☐ No ☐
Temp. ____ Vac. ____ Time ____
Rush Service ☐ Rush service guarantees analyses will be completed and results available by 5 PM EST on the day the sample is received by 11 AM.
Include Email Address or FAX # Below
xyang20@gsu.edu

Date Received MAY 21 2024 Date Completed MAY 22 2024
Remarks:

(2) HPLC trace of QCP-105

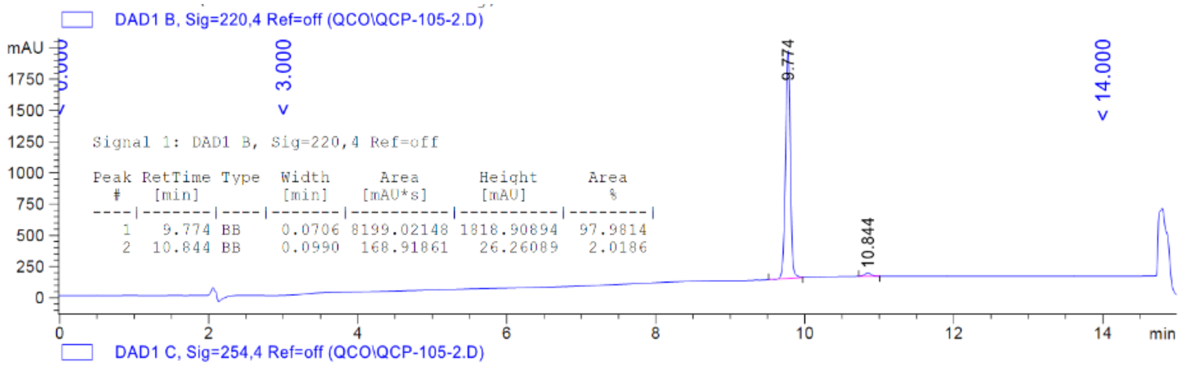

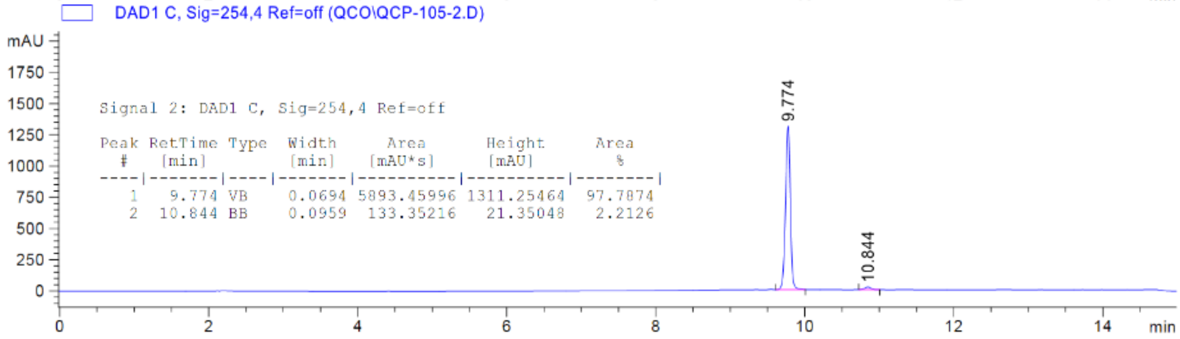

Solvent blank:

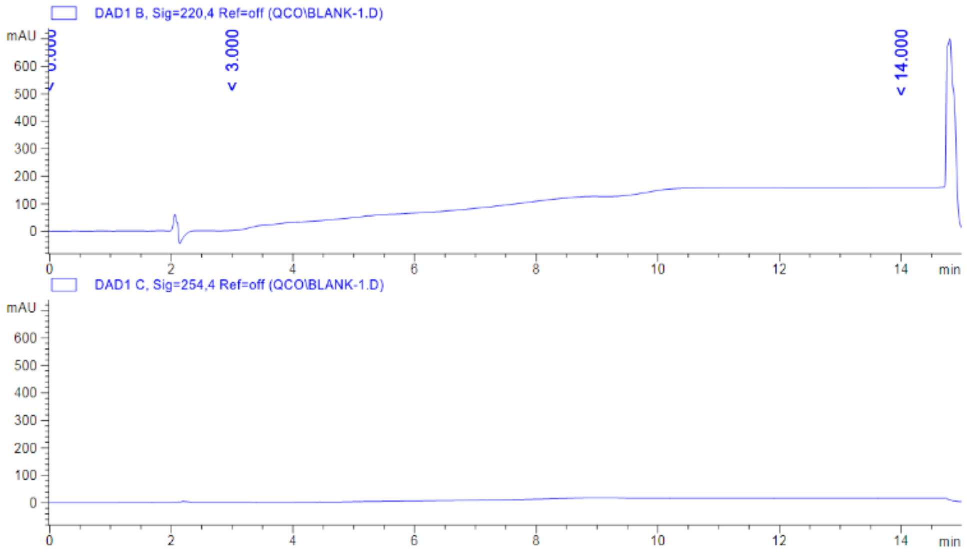

HPLC condition: Agilent 1100 HPLC with Kromasil C18 5 µm, 4.6×150 mm column; mobile phase: A: H_2O (0.1% TFA), B: ACN (0.1% TFA), gradient: 0 min 5%B -> 5 min 95% B -> 10 min 95% B -> 12 min 5% B -> 15 min 5% B, flow rate 1 mL/min; detector: DAD (monitored at 220 nm and 254 nm). Sample prepared in ACN; injection volume: 10 µL.

7. References

1. Huynh, M. T.; Anson, C. W.; Cavell, A. C.; Stahl, S. S.; Hammes-Schiffer, S., Quinone 1 e(-) and 2 e(-)/2 H(+) reduction potentials: identification and analysis of deviations from systematic scaling relationships. *J Am Chem Soc* **2016,** *138* (49), 15903-15910.
2. Wang, H.; Emanuelsson, R.; Banerjee, A.; Ahuja, R.; Strømme, M.; Sjödin, M., Effect of cycling ion and solvent on the redox chemistry of substituted quinones and solvent-induced breakdown of the correlation between redox rotential and electron-withdrawing power of substituents. *J Phys Chem C* **2020,** *124* (25), 13609-13617.
3. Yang, X.; Yuan, Z.; Lu, W.; Yang, C.; Wang, M.; Tripathi, R.; Fultz, Z.; Tan, C.; Wang, B., De novo construction of fluorophores via CO insertion-initiated lactamization: a chemical strategy toward highly sensitive and highly selective turn-On fuorescent probes for carbon monoxide. *J Am Chem Soc* **2023,** *145* (1), 78-88.
4. Michel, B. W.; Lippert, A. R.; Chang, C. J., A reaction-based fluorescent probe for selective imaging of carbon monoxide in living cells using a palladium-mediated carbonylation. *J Am Chem Soc* **2012,** *134* (38), 15668-71.
5. Von Hoff, D. D.; Layard, M. W.; Basa, P.; Davis, H. L., Jr.; Von Hoff, A. L.; Rozencweig, M.; Muggia, F. M., Risk factors for doxorubicin-induced congestive heart failure. *Ann Intern Med* **1979,** *91* (5), 710-7.
6. Lang, R. M.; Bierig, M.; Devereux, R. B.; Flachskampf, F. A.; Foster, E.; Pellikka, P. A.; Picard, M. H.; Roman, M. J.; Seward, J.; Shanewise, J. S.; Solomon, S. D.; Spencer, K. T.; Sutton, M. S.; Stewart, W. J.; Chamber Quantification Writing, G.; American Society of Echocardiography's, G.; Standards, C.; European Association of, E., Recommendations for chamber quantification: a report from the American Society of Echocardiography's Guidelines and Standards Committee and the Chamber Quantification Writing Group, developed in conjunction with the European Association of Echocardiography, a branch of the European Society of Cardiology. *J Am Soc Echocardiogr* **2005,** *18* (12), 1440-63.
